# Sickle Cell Disease in Children and Adolescents: A Review of the Historical, Clinical, and Public Health Perspective of Sub-Saharan Africa and Beyond

**DOI:** 10.1155/2022/3885979

**Published:** 2022-10-08

**Authors:** Walufu Ivan Egesa, Gloria Nakalema, William M. Waibi, Munanura Turyasiima, Emmanuel Amuje, Gloria Kiconco, Simon Odoch, Patrick Kumbowi Kumbakulu, Said Abdirashid, Daniel Asiimwe

**Affiliations:** ^1^Department of Pediatrics, Nile International Hospital, Jinja District, Uganda; ^2^Department of Pediatrics & Child Health, Faculty of Clinical Medicine & Dentistry, Kampala International University, Bushenyi District, Uganda; ^3^Department of Pediatrics, Luweero Hospital, Luwero District, Uganda; ^4^Standards Compliance Accreditation and Patient Protection (SCAPP) Department, Governance and Regulation Directorate, Ministry of Health, Kampala, Uganda; ^5^Department of Pediatrics, Fort Portal Regional Referral Hospital, Kabarole District, Uganda; ^6^Department of Surgery, Faculty of Clinical Medicine & Dentistry, Kampala International University, Bushenyi District, Uganda; ^7^Department of Surgery, Holy Family Virika hospital, Kabarole District, Uganda

## Abstract

Sickle cell disease (SCD) is an umbrella term for a group of life-long debilitating autosomal recessive disorders that are caused by a single-point mutation (Glu→Val) that results in polymerization of hemoglobin (Hb) and reversible sickle-shape deformation of erythrocytes. This leads to increased hemolysis of erythrocytes and microvascular occlusion, ischemia-reperfusion injury, and tissue infarction, ultimately causing multisystem end-organ complications. Sickle cell anemia (HbSS) is the most common and most severe genotype of SCD, followed by HbSC, HbS*β*^0^thalassemia, HbS*β*+thalassemia, and rare and benign genotypes. Clinical manifestations of SCD occur early in life, are variable, and are modified by several genetic and environmental factors. Nearly 500 children with SCD continue to die prematurely every day, due to delayed diagnosis and/or lack of access to comprehensive care in sub-Saharan Africa (SSA), a trend that needs to be urgently reversed. Despite proven efficacy in developed countries, newborn screening programs are not universal in SSA. This calls for a consolidated effort to make this possible, through the use of rapid, accurate, and cheap point-of-care test kits which require minimal training. For almost two decades, hydroxyurea (hydroxycarbamide), a century-old drug, was the only disease-modifying therapy approved by the U.S. Food and Drug Administration. Recently, the list expanded to L-glutamine, crizanlizumab, and voxelotor, with several promising novel therapies in the pipeline. Despite its several limitations, hematopoietic stem cell transplant (HSCT) remains the only curative intervention for SCD. Meanwhile, recent advances in gene therapy trials offer a glimpse of hope for the near future, although its use maybe limited to developed countries for several decades.

## 1. Background

Sickle cell disease (SCD) was first described in the western literature by James B. Herrick in 1910 [1]. He reported peculiar-looking red blood cells (RBCs) for which he had no definite diagnosis at the time. Herrick was alerted about this odd finding by his 27-year-old intern, Ernest E. Irons, who while based in Presbyterian Hospital, had observed pear-shaped and elongated cells in the blood smear of a 20-year-old negro named Walter Clement Noel. Noel, who was considered by Herrick as “bright and intelligent” was pursuing dentistry at Chicago College of Dental Surgery. Curious to establish the diagnosis, Herrick and Irons continued their follow-up of Noel for 2.5 years after this discovery, a period during which he suffered several severe illnesses. At 32 years, Noel died from pneumonia, 9 years after returning home to Grenada in West Indies [1, 2]. It was after this study that scientists began to decipher the molecular basis and pathophysiologic mechanisms of SCD. Long before Herrick's report, Horton had identified a clinical phenotype of SCD in the tropics [3]. SCD was described under different names in West Africa, including “Abiku,” “Ogbanje,” and “Sankara-jimi” and was treated using various traditional herbs [4, 5]. Similarly, SCD was well recognized by communities in East Africa as early as the beginning of the 18th century, where it was referred to as “Kagenge” and later “Nnalubiri” by Baganda in present day Uganda. Traditional healers observed characteristic presentations such as recurrent episodes of fever and pain, poor weight and height gain, chronic wounds, and early death; and unsuccessfully attempted to cure the disease using extracts from selected tree leaves and bark [6].

SCD was only recognized as a public health priority by the World Health Organization (WHO) in 2006 [7] and United Nations (UN) in 2008 [8]. In an effort to increase public knowledge and awareness about SCD, the UN recognized June 19 as the World Sickle Cell Day, which was first commemorated in 2009 [9]. SCD is predominant among people of sub-Saharan Africa (SSA), Mediterranean, Middle East, and Indian descent [10]; and has been disseminated across the globe through population migration [11]. Reliable estimates of the global population of individuals with SCD are not currently available, but some authors estimate it to be between 20 and 25 million [12]. More than 312,000 neonates are born with sickle cell anemia (SCA) annually, and 64 to 75% of these are in SSA where 50 to 90% of disease-related deaths occur in childhood [13, 14]. SCD is the most prevalent genetic disorder in the sub-Saharan region of Africa, with approximately 1% to 3% of newborns affected, the highest number of births being in the Democratic Republic of Congo, Nigeria, Tanzania, and Uganda [13–18]. Although projections show that the population of newborns with SCD is rising [14], a large fraction of SCD-related deaths in Africa occur prior to diagnosis, due to the absence of large-scale newborn screening programs [15, 19]. It is unfortunate that in SSA, nearly 500 children aged less than five years die every day due to SCD-related complications [20], with SCD-associated mortality accounting for almost 9-16% of under-five mortality in high-burden countries [21]. Conversely, more than 93% of patients with SCD in developed countries live into adulthood [22], with a median survival age between 58 and 66 years [23].

Despite the fact that many countries in SSA have shown interest in SCD, national policies and funding are still inadequate, mingled with inadequately trained healthcare providers, scarce diagnostic tools, and insufficient treatment [7]. The resultant poor health status and multiple hospitalizations of children with SCD tremendously impact on their quality of life and academic achievements, parents' employability, and productivity, and worsen the already poor socioeconomic and psychosocial burden on families in high-burden countries [20].

In this integrated review, we aimed to (1) summarize the pathogenesis and genotypes of SCD, (2) clinical manifestations and complications of SCD in the pediatric population, (3) summarize the comprehensive care package for SCD, (4) underscore the current state of NBS in sub-Saharan Africa and performance of point-of-care tests, (5) highlight evidence of the beneficial effects of currently approved disease-modifying therapy and pipeline drugs, (6) adolescent to adult transition, and (7) prevention of SCD.

## 2. Myths and Misconceptions about SCD in SSA

Several myths and misconceptions are known to influence people's understanding and care of SCD in Africa and beyond. According to the cultural beliefs of the Igbo in Nigeria, children who are currently defined as those with SCD were conceptualized as those who mysteriously experienced repetitive cycles of birth, death, and reincarnation. They often gave family names that are related to death and performed various rituals after the death of an infant or young child in an effort to stop the reoccurrence of reincarnation [5]. Literature shows that some African communities still believe that SCD is caused by evil spirits/witchcraft or misconduct [4, 24]. In a recent Tanzanian study, participants believed that SCD is for the poor and is a punishment from God [25]. Some men believe that males do not carry the sickle cell gene. Zounon [26] pointed out that lay people in Benin are not sufficiently aware that SCD is inherited; can be detected via genetic testing; does not automatically result in illness; cannot be cured by traditional healers; and that SCD may induce severe kidney, lung, heart, or cerebrovascular complications. Others believe that SCD only affects people of black race; it is blood-borne and contagious; cannot be cured; affected babies do not make it to adulthood; and affected individuals are addicted to pain medication. In addition, some people think that the sickle cell trait can convert into SCD and people with SCD do not suffer from malaria [27, 28]. With this in mind, we strongly recommend that clinicians and concerned stakeholders such as sickle cell foundations and Ministries of Health scale up efforts to continuously sensitize the public about the facts regarding SCD, because these numerous misconceptions adversely influence health-seeking behavior.

## 3. Structure and Function of Normal Hemoglobin

The hemoglobin molecule is a metalloprotein composed of four subunits, each containing a peptide chain and a heme group. The polypeptide chains of hemoglobin are symmetrically paired to form a tetrameric structure and functional unit. Each of the four polypeptides has a large central space into which a heme prosthetic group and an iron protoporphyrin IX molecule are bound by noncovalent forces, thus protecting the iron atom (Fe2+) from access to the surrounding aqueous solution. The alpha (*α*) polypeptide chains and heme group iron protoporphyrin IX are the same in all human hemoglobins. The arrangement of the subunits of hemoglobin (quaternary structure) differs in the oxygenated and deoxygenated states. Oxygen reversibly binds to the ferrous iron atom in each heme group. When oxygen molecules attach to the ferrous iron atoms, the gap between two of the polypeptide chains in the hemoglobin molecule becomes narrower, expanding when oxygen leaves. Besides the transport of oxygen from the lungs to tissues, hemoglobin also interacts with carbon dioxide, carbon monoxide, and nitric acid [29, 30].

Three *α* gene clusters (zeta - *ζ*, alpha 1 - *α*_1_, and alpha 2 - *α*_2_) and five *β* gene clusters (epsilon - *ε*, gamma 1 - *γ*_1_, gamma 2 - *γ*_2,_ delta - *δ*, and beta - *β*) are found at the p13.3 locus of chromosome 16 (16p13.3) and p15.5 locus of chromosome 11 (11p15.5), respectively. During fetal life, Gower 1 (*ζ*_2_*ε*_2_), Gower 2 (*α*_2_*ε*_2_), and Portland (*ζ*_2_*γ*_2_) hemoglobins are formed because their genes are expressed primarily in the yolk sac, para-aortic region, and the liver. Their downregulation in early embryonic life is followed by the expression of two *α* and two *γ* genes, leading to the formation of fetal hemoglobin (*α*_2_*γ*_2_), which is the predominant hemoglobin by the 9th week of gestation. Fetal hemoglobin (HbF) has a slightly higher affinity for oxygen than adult hemoglobin because it binds 2,3-biphosphoglycerate (2,3-BPG) less strongly. After birth, the *α* genes remain fully active, whereas *γ* genes are downregulated, and the *β* cluster genes (*β* and *δ*) are upregulated. The adult hemoglobin phenotype is predominant by the end of the first 12 months of life. Normal adult human RBCs contain HbA (*α*_2_*β*_2_), HbA2 (*α*_2_*δ*_2_), and HbF (*α*_2_*γ*_2_), accounting for nearly 97%, 2%, and 1% of the total hemoglobin respectively. In rare cases, however, *γ*-globin gene expression persists in adult RBCs, a condition referred to as hereditary persistence of fetal hemoglobin (HPFH) [29, 30].

## 4. Genotypes and Pathophysiology of SCD

**Figure 1:**
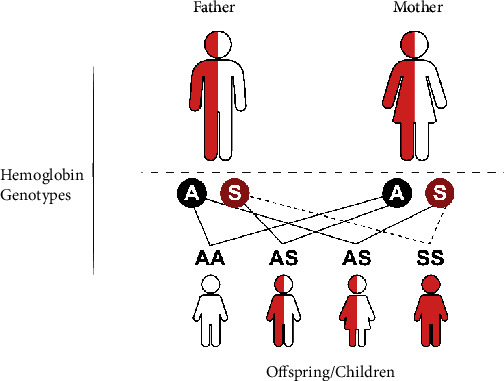
Inheritance of sickle cell disease. In a scenario where both parents have a sickle cell trait (SCT, HbAS), each pregnancy carries a 25% chance of normal offspring (HbAA), a 50% chance of offspring with SCT, and a 25% chance of offspring with sickle cell disease (HbSS).

Sickle cell disease is an umbrella terminology that describes a group of life-long debilitating autosomal recessive disorders which occur as a result of a single-point mutation (base change from GAG to GTG at codon 6, rs334) where a hydrophobic valine replaces hydrophilic glutamic acid at position 6 on the *β*-globin subunit of hemoglobin (c.20A>T, pGlu6Val) [31–33]. This mutation leads to the formation of an abnormal form of hemoglobin designated as sickle hemoglobin (HbS). Classically, SCD follows an autosomal recessive Mendelian pattern of inheritance where an affected offspring (male or female) receives one defective gene from each parent, as illustrated in Figure 1.

There are variable genotypes of SCD, but the most common is sickle cell anemia (HbSS), which results from inheritance of two copies of the HbS mutation (homozygotes), and accounts for 65% to 70% of all cases of SCD. Other common forms of SCD include hemoglobin SC (HbSC), sickle hemoglobin-beta-zero thalassemia (HbS*β*^0^thalassemia), and sickle hemoglobin-beta-plus thalassemia (HbS*β*+thalassemia) which occur when there is coinheritance of mutations responsible for other abnormal types of hemoglobin (i.e., HbC or *β*-thalassemia) combine with HbS forming a compound heterozygous mutation [32, 34, 35]. Patients with HbS*β*+thalassemia have reduced levels of *β*-globin production, while those with HbS*β*^0^thalassemia have no *β*-globin production [32]. HbC is caused by glutamic acid replacement by lysine at the same site as that for HbS (c.19G>A, p.Glu6Lys) [33, 36]. Other *β*-globin gene mutations include HbD Punjab/Los Angeles (c.364G>C, p.Glu121Gln), HbE, Hb-Lepore, HbO-Arab (c.364G>A, p.Glu121Lys), and Hb Quebec-CHORI, among others [33, 35, 37, 38]. Coinheritance of HbS and these hemoglobin variants leads to rare and relatively benign SCD genotypes (HbSD-Punjab/Los Angeles, HbSE, HbS-Lepore, and HbSO-Arab, HbS-Quebec-CHORI) [39].

**Figure 2:**
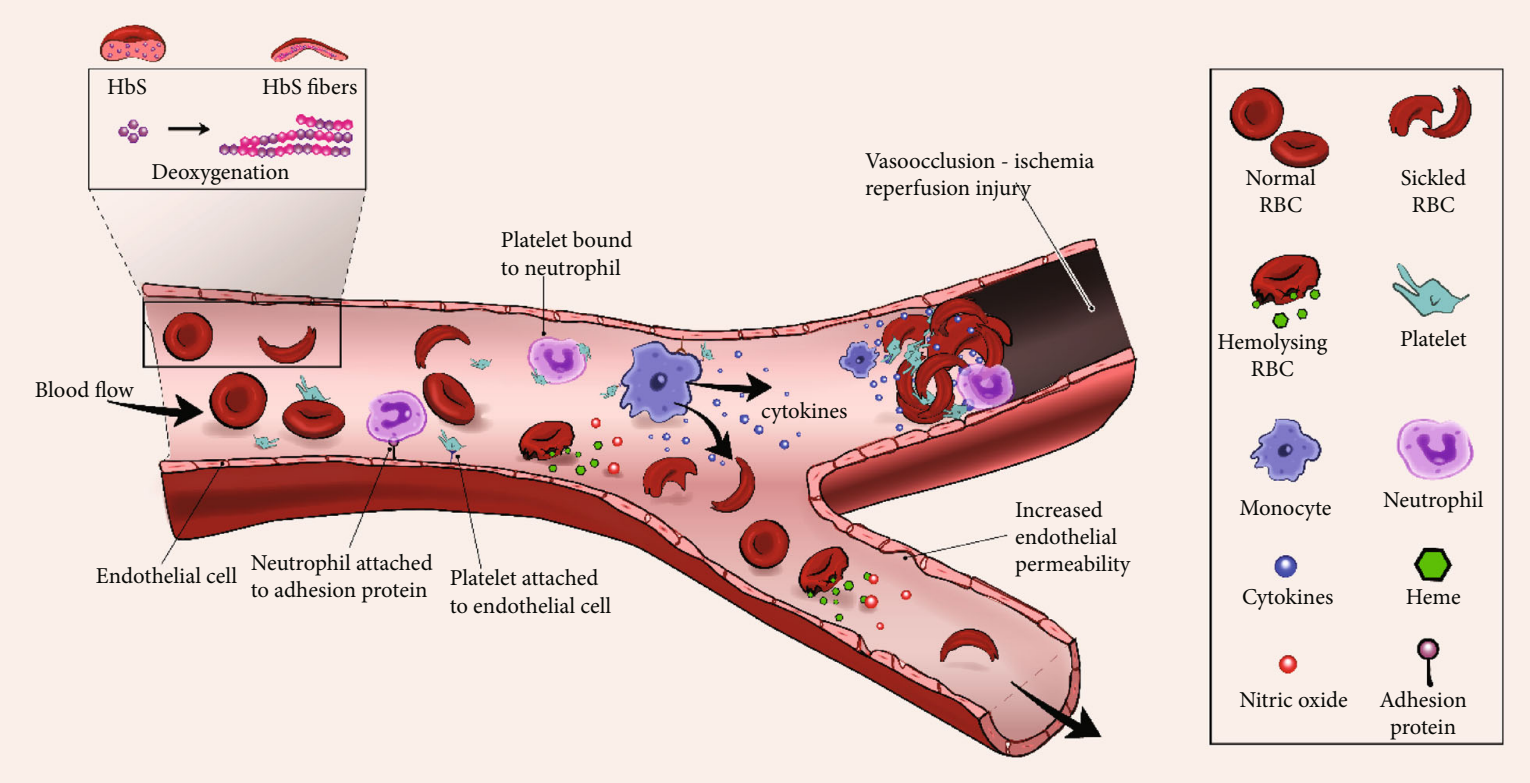
Pathophysiologic mechanisms of sickle cell disease. Deoxygenation leads to polymerization of HbS and deformation of RBCs to a sickle shape, making them less flexible. Broadly, RBC, leucocyte, and platelet adhesion are enhanced, and cell-to-cell and cell-to-endothelial interactions occur; there is an upregulation of adhesion proteins (ICAM-1, VCAM-1, P-selectin, and E-selectin); and damage and activation of the endothelium occur. RBC hemolysis releases heme and arginase which scavenge nitric oxide, and stimulates the release of adhesion proteins. Activated platelets cause neutrophils to form neutrophil extracellular traps (NETs), which induce platelet and RBC aggregation; interact with sickled RBCs, neutrophils, and monocytes. Neutrophils also adhere to the endothelium through interaction with adhesion proteins. Monocytes release cytokines, platelet-activating factor, and express tissue factor which upregulate endothelial adhesion proteins, activate platelets, and kickstart the coagulation cascade. Accumulation of leukocytes, platelets, and RBCs with coagulation pathway activation through various mediators leads to the formation of thrombi which are responsible for vaso-occlusion. These events subsequently cause local ischemia and reperfusion injury which leads to organ dysfunction.

Deoxygenation results in intracellular polymerization of molecules of HbS into rigid crystal-like rods which deform the normally flexible biconcave RBCs into the characteristic rigid sickled (crescent) shape [36]. Although polymerization is reversible with reoxygenation, repetitive episodes cause RBC membrane damage and hemolysis [31], reducing the lifespan of RBCs from the normal 90-120 days to 10-20 days [40]. More rapid polymerization occurs with higher concentrations of HbS and low pH but is limited by the presence of HbF. The hallmark pathophysiologic mechanism is the entrapment of sickled RBCs in the microvasculature and the impediment of blood flow, leading to typical acute and chronic ischemic multiorgan complications [31, 34]. Mechanisms are complex and not yet completely understood, but abnormal RBC adhesion, activation of the vascular endothelium, leukocytosis, leukocyte (neutrophils and monocytes) and platelet activation, cellular dehydration, and oxidative stress from tissue reperfusion have been implicated [36, 41, 42]. Molecules such as P-selectin, E-selectin, intercellular adhesion molecule-1 (ICAM-1), vascular cellular adhesion molecule-1 (VCAM-1), laminin, and thrombospondin are critical contributors in adhesion [42–44]. This is schematically represented in Figure 2. Increasing knowledge of the pathophysiologic mechanisms that culminate in sickling and subsequent processes has been key in the design of new therapies. Research focus has been set on haptoglobin and hemopexin for their role in binding free extracellular Hb and heme, respectively, HbF-inducing agents (e.g., hydroxyurea, voxelotor), selectin inhibitors (e.g., crizanlizumab), anti-inflammatory agents, antiplatelet agents, anticoagulants, and antioxidants (e.g., L-glutamine) [42, 45–47].

## 5. Sickle Cell Trait

Sickle cell trait (SCT), not categorized as a SCD, refers to a state where individuals are heterozygous for the sickle allele (HbAS, also known as carriers). It results from inheritance of one abnormal (HbS) allele from one parent and one normal (HbA) allele from another parent [48]. One in every five people has SCT in high-burden countries like Nigeria [49].

Earlier studies characterizing the DNA structure of the *β*-globin locus of HbS suggest that the mutation arose on at least three independent occasions in the African continent, referred to as *β*-globin haplotypes. These haplotypes are determined by restriction fragment length polymorphism (RFLP) and were named after the ethnic group or geographic area where they were first described, namely, Benin (BEN), Senegal (SEN), Cameroon (CAM), Central African Republic (CAR) or Bantu, and Arab-Indian (AI) haplotypes [50]. Atypical haplotypes have been described in an ethnically diverse Sudanese population with SCA [51]. Results of whole-genome sequencing reveal that the sickle allele had a single origin 259 generations back (7,300 years ago) in West Africa during the Holocene Wet Phase [50]. The distribution of the sickle allele correlates with the distribution of malaria [52]. Worldwide, nearly 5.5 million neonates are born with SCT annually, 65% of them in the WHO African region, where malaria is highly prevalent [14]. SCT confers a significant survival advantage in regions of high malaria endemicity. A 2014 multicenter study of 11,890 cases of severe *Plasmodium falciparum* malaria and 17,441 controls recruited from 12 locations in Africa (The Gambia, Mali, Burkina Faso, Ghana Navrongo and Kumasi, Nigeria, Cameroon, Kenya, Tanzania, and Malawi), Asia (Vietnam), and Oceania (Papua New Guinea) found that people with HbAS have an 86% lower risk of developing severe malaria [53]. Individuals with SCT are usually clinically asymptomatic [34] and have a normal life expectancy similar to unaffected people, but are not necessarily free of adverse outcomes. Accumulated evidence shows that they have a high risk of vaso-occlusive pain, hyposthenuria, proteinuria, hematuria, renal medullary carcinoma, venous and pulmonary thromboembolism, and rhabdomyolysis following extreme physical exertion [36, 54–56]. Nonetheless, these events are rare.

## 6. Newborn Screening for SCD in Sub-Saharan Africa

### 6.1. Current State of NBS Programs in SSA

Early diagnosis and initiation of comprehensive care for genetic diseases like SCD can be life-saving. This can be achieved prenatally through chorionic villus sampling or amniocentesis, or postnatally through newborn screening [32]. Although universal NBS programs have been successful in developed regions such as Europe [35, 57] and North America [58], this has not been prioritized by countries in sub-Saharan Africa where many of the targeted NBS initiatives are still in infancy, mostly limited to feasibility and pilot studies, and in a few centers [18, 59–61]. Universal NBS has not been widely implemented even in Ghana [17] and Benin [59] where substantial progress in NBS research capacity and follow-up has been documented since the early 90s. In Uganda, screening for SCD is focused on children aged 24 months or less, and in districts with the highest burden of SCD [18]. Certainly, we need to acknowledge the numerous limitations to the expansion of the program in SSA [62].

Mcgann [15] in his advocacy for countries to prioritize SCD suggests that NBS programs in Africa can sustainably be integrated into already existing infrastructure such as the expanded program of immunization and the HIV early infant diagnosis (HIV-EID) at a low cost. A notable example is the study by Ndeezi et al. [63] which determined the prevalence of sickle cell trait and SCD using dried blood spots that are routinely collected from HIV-exposed infants across Uganda. In Nigeria, Nnodu and colleagues [16] demonstrated that NBS can be feasibly implemented through integration into the routine immunization structure with minimal additional human resource and finances. In Ghana, newborns are screened for SCD at birth, at well-infant clinics, or within a few weeks after birth [17]. Newborns delivered outside health facilities can be screened during their first vaccination. However, there is a need to raise public awareness about the relevance of newborn screening and early interventions through concerted efforts by Ministries of Health and partners [49, 64].

An innovative collaboration between the American Society of Hematology (ASH), hematologists and public health specialists in SSA, and the Consortium on Newborn Screening in Africa (CONSA) was launched in 2020 to demonstrate the benefits of NBS and early access to clinical interventions at participating institutions in 7 countries, including Kenya, Ghana, Liberia, Nigeria, Uganda, Tanzania, and Zambia [64]. The pilot project aims to screen 10,000 to 16,000 babies per year in each country for five years, while providing clinical follow-up for newborns with SCD [64]. However, the sustainable implementation of such major projects in low- and middle-income countries following the withdrawal of major funding institutions remains a key challenge, often driven by health priorities and funding.

### 6.2. The Value of Point-of-Care Testing for NBS

Conventional methods for performing NBS such as hemoglobin electrophoresis, isoelectric focusing (IEF), DNA testing, and high-performance liquid chromatography (HPLC) require expertise, expensive equipment, reagents, electricity, and long-distance transportation of blood specimens and laboratory results [18, 65]. Wide-scale implementation is also challenging in low-resource countries because of the high turnaround time for results, and yet, locating the family afterward is often difficult [65]. In many health facilities in Africa, the sickling test and sickle solubility test are readily used to screen for SCD. However, these tests do not differentiate SCT from SCD [66]. Using standard methods such as hemoglobin electrophoresis makes it possible to differentiate hemoglobinopathies in the newborn, based on the Hb types present. Normal screening results show HbF and HbA (F, A). Sickle cell trait (F, A, S); HbSS, HbS-*β*^0^thalassemia, or HbS-HPFH (F, S); HbSC (F, S, C); HbS*β*+thalassemia (F, S, A); SCT with *α*-thalassemia trait (F, A, S, Barts); and *β*-thalassemia major (F) can be diagnosed [32].

A rapid, easy to use, accurate, and cheap point-of-care test (POCT) for SCA is key in making widespread screening possible and efficient [15]. A pilot study of newborns and infants younger than 9 months who presented for immunization at five primary health facilities in Nigeria showed that POCTs are accurate and reliable [16]. By using two lateral flow immunoassay POCTs (HemoTypeSC™ and Sickle SCAN™), both tests had a specificity of 100% and a sensitivity of 100% for detection of SCD when compared to high-performance liquid chromatography as a gold standard. An earlier multicenter study involving 1,121 neonates and infants at 18 health facilities in Nigeria found a 93.4% sensitivity and 99.9% specificity of HemoTypeSC™ rapid test kit for the diagnosis of SCA, with an overall accuracy of 99.1% in field conditions [67]. In a study conducted across three study sites in Ghana, Martinique, and USA, HemoTypeSC™ had an overall sensitivity and specificity of 99.5% and 99.9%, respectively across all hemoglobin phenotypes, 100% sensitivity and specificity for SCA, and >99% sensitivity and specificity for detection of normal and trait states [68]. Similar results were documented by researchers using HemoTypeSC™ in Nigeria [69] and Côte D'Ivoire [70], and using Sickle SCAN™ in Mali and Togo [71].

POCTs require only 1 to 5 *μ*L of blood from a finger prick. POCTs identify HbA, HbS, and HbC, and can therefore diagnose the HbAS, HbSS, HbSC, and HbCC phenotypes; but they cannot quantify different hemoglobins or accurately identify other forms of SCD such as HbS*β*-thalassemia [72]. The cost of one POCT ranges from US$1.49 to US$5.09, with results available in approximately 10 minutes through visual inspection of the test line color intensity [16, 68, 73]. Moreover, the test requires minimal operator training, with no need for electricity, refrigeration, or other instruments [68]. Unfortunately, these tools have not translated into national NBS programs, as many nationwide NBS programs are pilot projects.

## 7. Helping Families Cope with a Diagnosis of SCD

It is not uncommon for health practitioners to slap a new diagnosis without due consideration of the emotional distress and the feeling of guilt that parents experience. Many parents in Africa have limited knowledge about SCD and thus view it through socio-cultural, religious, and economic dimensions. Some communities believe that SCD is a curse to their families and worry about social stigma and medical expenses [74, 75]. Individuals with SCD are perceived to be witches and wizards. Some parents pay less attention and invest less because they link the diagnosis to early child mortality and “recurrent” death, which undermines their survival [75]. Parents may initially deny the diagnosis and fail to comply with medical recommendations. Fathers may deny paternity, accuse women of sexual unfaithfulness, and blame them for being responsible for SCD, resulting in family separation [76]. Therefore, a face-to-face interaction is crucial when announcing the results, considering the importance of pretest and post-test counselling [72]. Parents need support to focus on the expected achievements and future personal and professional life of their children. This can be achieved by thorough education of parents about SCD (genetics, transmission, and care), sharing experiences among affected families, provision of psychological support [65], and linkage to a sickle cell treatment center.

## 8. Clinical Presentation of SCD

Clinical manifestations of SCD occur at a time when *γ*-globin chain synthesis decreases and is replaced by *β*-globin chain synthesis. This leads to decreased production of HbF and increased production of HbS, at a transition rate that varies among individuals [48]. Typically, HbF levels drop during the first few months of life from nearly 85% of the total hemoglobin at birth to less than 1% at 12 months of life, accompanied by increased synthesis of HbA in normal individuals [77]. Individuals with HbSS and HbS*β*^0^thalassemia have the most severe clinical and hematologic phenotype, whereas those with HbSC and HbS*β*+thalassemia have a less severe spectrum of manifestations. The baseline hemoglobin level for patients with HbSS and HbS*β*^0^thalassemia ranges from 6 to 9g/dl, while that of HbSC and HbS*β*+thalassemia ranges from 9 to 11g/dl and 10 to 12g/dl respectively [32]. For unknown reasons, patients with HbSC and HbS*β*+thalassemia are more prone to proliferative sickle retinopathy which can lead to loss of vision [48, 78]. Our discussion will mainly focus on SCA because it is the most prevalent and most severe form of SCD in SSA.

Individuals with SCA experience numerous multisystemic complications (Table 1). Infants with SCA may present with dactylitis (hand-foot syndrome) and splenic sequestration as early as 3 months of life [48]. Dactylitis affects up to 45% of children by 2 years of life [79], becoming rare after 5 years of life. Other common manifestations include jaundice, anemia, acute chest syndrome (ACS), and overwhelming infections. Children experience acute and chronic pain of varying intensity, jaundice, anemia, splenic sequestration, ACS, stroke, infections, poor nutritional status, poor academic performance, and delayed puberty [34, 48], among other complications [80] (Tables 1–4). Adolescents exhibit enuresis, priapism, chronic leg ulceration, increased vaso-occlusive bone pain episodes, avascular necrosis of the hip, and delayed physical growth and puberty, which require significant clinical and psychosocial support [48]. The most common reasons for hospitalization include vaso-occlusive pain, severe anemia, infections, stroke, and acute chest syndrome [81, 82]. Triggers of crises range from dehydration to cold exposure, physical exertion, psychological stress, injury (e.g., following surgery), infections or infestations, pregnancy, smoking, and drugs, among others [24, 40, 49].

## 9. Predictors of SCD Severity

Genetic and environmental modifiers have been found to result in considerable variability of clinical phenotypes, given that some individuals experience milder symptoms and live longer, whereas others live with frequent and severe complications and premature mortality [84, 85]. In as much as SCD has been well studied, the complex effect of genetics on disease phenotype is yet to be deeply understood and may provide an additional window for targeted disease-modifying treatment and prognostication [46, 86].

A study of 9 identical twins and 350 age-gender-matched sibling pairs in Jamaica found no significant differences among identical twins with SCD regarding attained height for age, weight for height, HbF levels, total Hb, mean cell volume, mean cell hemoglobin and total bilirubin levels, occurrence and grade of splenomegaly, susceptibility to priapism, and age at menarche. The occurrence and severity of other complications (e.g., acute chest syndrome, aplastic crisis, cerebrovascular events) were variable, suggesting the contribution of nongenetic (environmental) factors [87].

### 9.1. Environmental Factors

The contribution of factors such as environmental temperature, air quality, bacterial and viral infections, malaria, nutritional status, socioeconomic status, physical activity, and public health measures (e.g., immunization) on the clinical expression and outcome of SCD cannot be underestimated [48, 88]. More prospective studies need to be conducted in this area.

Available evidence shows that cigarette smoking, whether active or passive smoke exposure, results in increased hospitalization for vaso-occlusive pain and acute chest syndrome [89, 90]. Components of cigarette smoke displace oxygen from hemoglobin, damage the vascular endothelium, and lead to platelet aggregation and thrombus formation [90]. This data suggests that decreasing tobacco smoke exposure for children with SCD could lower morbidity, mortality, and medical expenses [90]. Unfortunately, this concept is not addressed in sickle cell treatment guidelines.

### 9.2. Genetic Factors

*Fetal hemoglobin:* HbF is probably the most extensively studied modulator of SCD severity. Patients with SCA have HbF concentrations ranging from 0.1% to 30%, with an average of 8% [86]. Children with higher HbF levels are less likely to be hospitalized [82]. Hereditary persistence of fetal hemoglobin is a rare benign disorder which presents with high HbF levels of approximately 30% that persists into adulthood. HPFH can be deletional or non-deletional and have a pancellular or heterocellular distribution. Tolu and colleagues [91] observed that individuals who coinherit HPFH and SCD have a delayed onset of severe complications, although this survival advantage attenuates with increasing age. There is no doubt that the polymerization-inhibiting effect of HbF depends on the distribution of HbF in RBCs [77]. Perhaps, a good predictor of disease severity is the distribution of HbF concentrations among F-cells (a subset of erythrocytes that contain HbF), and not total HbF or F-cell percentages [77, 92].

*α-thalassemia:* In the African population, *α*-thalassemia mainly occurs as a result of 3.7 kb *α*-globin gene deletions (^−3.7^*α*_2_/*α*_1_*α*_2_ or ^−3.7^*α*_2_/^−3.7^*α*_2_) [93, 94]. Co-existence of *α*-thalassemia in individuals with HbSS results in reduced mean corpuscular hemoglobin concentration (MCHC), reduced intracellular HbS concentration and HbS polymerization, lower number of irreversibly sickled RBCs, higher hematocrit, higher age at diagnosis, and fewer visits to the hospital [82, 94–96]. The median RBC count increases, whereas the median mean corpuscular volume (MCV) and WBC count significantly decrease with increasing number of *α*-globin gene deletions [94]. Through a study to determine the frequency of deletional *α*-thalassemia among blacks with SCA in the United States and Africa, Mears and colleagues [97] revealed a frequency of 0.12 to 0.16 in normal participants and 0.18 to 0.20 in those with SCT. The frequency was significantly high among patients with SCA, ranging from 0.22 to 0.33. There was a significant association between increasing age and the frequency of deletional *α*-thalassemia, implying that individuals with SCA and *α*-thalassemia live longer. This finding has been replicated by other researchers in Africa [94].

*HbS haplotypes:* Powars and Hiti [96] demonstrated that patients with CAR haplotype have the most severe progression of clinical manifestations and are more likely to die during the first three decades of life; those with the Senegal haplotype have less severe disease, whereas those with the Benin haplotype had intermediate severity. This is because individuals with Senegal and Benin haplotypes have high HbF levels compared to the CAR haplotype [96, 98, 99]. The Arab-Indian haplotype is associated with the highest HbF levels and frequent *α*-thalassemia, which inhibit sickling and ameliorate the clinical severity of the disease [48, 100].

## 10. Comprehensive Care of Children with SCD

### 10.1. Overview

The main goal of care is to improve the quality of life and life expectancy of individuals with SCD [24, 49]. This is achieved through coordinated care in community settings, primary care and specialist practices, emergency departments, laboratories, and sickle cell clinics in hospitals [83]. Comprehensive care of children with SCD includes simple proven measures such as patient/caregiver education on early identification of clinical presentations of life-threatening complications such as fever, severe anemia, and splenomegaly, pneumococcal, *Hemophilus influenza* type b (Hib) and meningococcal vaccines, prophylactic antimalarials and penicillin, analgesics, nutritional supplements, blood transfusion, disease-modifying drugs, and planned clinic evaluations [32, 34, 49, 65, 101, 102]. These measures can be categorized as supportive (balanced diet, folic acid, and hydration), preventive (chronic blood transfusion to prevent stroke, hydroxyurea, L-glutamine, crizanlizumab, voxelotor, penicillin prophylaxis, and pneumococcal vaccination), symptomatic (antibiotics, blood transfusion, and analgesics), and curative (hematopoietic stem cell transplant) approaches. Compliance to these measures results in improved physical growth, a remarkable reduction in the frequency and severity of acute events requiring hospitalization, and lower mortality [102]. A summary of recommended drugs and health monitoring of children with SCD is provided in Tables 2 and 3, respectively.  Table 4 summarizes the clinical manifestations, evaluation, and management of acute complications associated with SCD.

Major drawbacks to the implementation of comprehensive care measures in SSA include the existence of specialized care facilities in large cities [65], absence of comprehensive SCD treatment guidelines in many health facilities [103, 104], limited access to approved disease-modifying drugs, and ultimately, scarcity of health practitioners. It is obvious that up-to-date clinical protocols facilitate uniformity and standardization of care across different health facilities [49]. Until more recently, several high-SCD-burden countries lacked published SCD diagnosis and treatment guidelines. For instance, national guidelines for Nigeria were released in 2014 [49], whereas those of Uganda [24], Tanzania [105], and Kenya [72] were released in 2020. In as much as the recommendations in these guidelines are evidence-based, a very small fraction of health facilities in SSA have the necessary infrastructure and human resource to perform investigations such as transcranial Doppler ultrasonography (TCD) screening and extended phenotyping of blood to reduce the risk of alloimmunization [65], and neuroimaging. More worrying is the rarity of dedicated SCD care centers or clinics in the region. As such, children with SCD are queued in general clinics where they receive less attention. To facilitate improvement in SCD care in these settings, we suggest the development of handbooks [106] and their widespread distribution and clinician training of basic guidelines.

### 10.2. Malaria Chemoprophylaxis

Malaria chemoprophylaxis combined with mosquito net use, early diagnosis, and treatment are established preventive strategies in regions where malaria is highly prevalent. Sulfadoxine-pyrimethamine (SP), sulfadoxine-pyrimethamine + amodiaquine, pyrimethamine, proguanil, chloroquine, mefloquine, and mefloquine-artesunate are among the drugs that have been studied in sub-Saharan Africa [107]. Studies showed that monthly SP has greater potential to reduce episodes of malaria compared to weekly chloroquine [108] and daily proguanil [109]. Chemoprophylaxis reduces hospitalization rates, blood transfusion requirements, and symptoms such as vaso-occlusive pain [107, 110, 111], with good tolerability [107, 112].

### 10.3. Penicillin Prophylaxis and Pneumococcal Vaccination

Development of functional asplenia in children with SCA makes them susceptible to fulminant infection by encapsulated organisms, particularly *Streptococcus pneumoniae*, but also by *Hemophilus influenza type b* and *Neisseria meningitidis* [113]. Other organisms include *Escherichia coli*, *Staphylococcus species*, *Pseudomonas species*, *Salmonella species*, *Enterobacter species*, *Acinetobacter* species, and *Klebsiella species* [113, 114]. Results of a landmark study [115] showed that twice daily penicillin prophylaxis significantly reduces the incidence and mortality associated with *S. pneumoniae* septicemia. In this study, the incidence of pneumococcal bacteremia was 84% lower in the penicillin group compared with the placebo control group. These findings translated into the inclusion of Phenoxymethylpenicillin (Pen-V) in SCD treatment guidelines. However, the practice of penicillin prophylaxis in SSA is not strongly supported by evidence from the African region, given that recent studies show conflicting results regarding the predominance of *S. pneumoniae* as a cause of bacteremia in SCA [103, 116, 117]. This justifies the need to obtain high-quality evidence regarding the etiology of infection and develop appropriate preventive strategies for bacterial infections in children with SCD living in SSA.

Penicillin chemoprophylaxis should be started as soon as the diagnosis of SCA is made, preferably by 2 months of life [113], because the risk of bacteremia is highest in younger children. However, controversy still exists regarding the ideal timing of discontinuation of penicillin. To date, results of the PROPS II study published in 1995 [118] serve as a benchmark for the widespread practice of stopping prophylaxis after 5 years of life without increasing morbidity. Discontinuation of penicillin prophylaxis can be considered for children aged 5 years and over who have received the PCV13 and PPSV series, received prolonged periods of penicillin prophylaxis, are under regular medical supervision, have no history of severe pneumococcal infection, or have not had surgical splenectomy [113, 118]. However, penicillin prophylaxis can be continued indefinitely in those with recurrent invasive pneumococcal infections and those who have undergone splenectomy [83].

A major drawback has been the development of antimicrobial-resistant *S. pneumoniae*, but the nasopharyngeal carriage of antibiotic-resistant serotypes seems to have been abated by immunization [113, 118, 119]. Children with SCA should receive routine doses of PCV13, Hib, and meningococcal vaccines administered within a few weeks to a few months after birth. Pneumococcal polysaccharide vaccine (PPSV23) should be administered at 2 years and repeated at 5 years of life [49, 83, 113]. For adequate protection against invasive pneumococcal infections, parents and caregivers should be advised to ensure completion of the vaccine series prior to discontinuation of penicillin prophylaxis at 5 years [83].

### 10.4. Chronic Blood Transfusion

Long-term (chronic) transfusion therapy is recommended in primary and secondary prevention of stroke and is aimed at increasing the oxygen-carrying capacity and the proportion of HbA relative to HbS to prevent or reverse vaso-occlusion-related complications [120]. Approximately 10% of children with HbSS develop overt stroke in the absence of primary prevention [83]. Without monthly blood transfusion, 50% of children with a first stroke experience a second stroke within two years of the initial event [49, 121]. Adams and colleagues [122] demonstrated that the risk of a first stroke in children with abnormal TCD (≥200 cm/sec) is reduced by 92% if monthly blood transfusion is provided. Chronic transfusion therapy leads to normalization of TCD velocity in children with HbSS or HbS*β*^o^ thalassemia. Results of the STOP 2 trial [123] showed that discontinuation of chronic blood transfusion for prevention of primary stroke in children leads to a high rate of reversal to abnormal TCD blood velocities and risk of stroke.

Blood transfusion can be performed as simple transfusion, manual exchange transfusion, or automated red blood cell transfusion [83, 123]. The advantage of exchange transfusion over simple transfusion is that it reduces HbS level and it results in lower risk of hyperviscocity and lower levels of iron accumulation [120]. Children with acute ischemic stroke should receive EBT with a target Hb of 10 g/dL and HbS <30%. Meanwhile, preoperative simple blood transfusion targets Hb 10 g/dL if general anesthesia is to be used [83]. Individuals receiving regular blood transfusion should have their iron status monitored and iron chelation started using Desferrioxamine (DFO), Deferiprone (DFP), Deferasirox (DFX), or a combination of DFO and DFP when the serum ferritin concentration is ≥1000 *μ*g/L or after 10-20 blood transfusions [49, 72]. Another complication of blood transfusion is alloimmunization, which can be minimized by matching donor blood and patient blood not only for ABO antigens, but also performing extended phenotyping for C, E, K, S, and s antigens [46, 83]. Unfortunately, extended phenotyping and screening for alloantibodies are rarely performed in SSA; a region where shortage of packed RBCs and leucocyte-depleted blood is commonly observed [124]. Besides, accurate diagnostic methods for iron overload are lacking, and treatment is costly and not readily available [124].

### 10.5. Hydroxyurea

**Figure 3:**
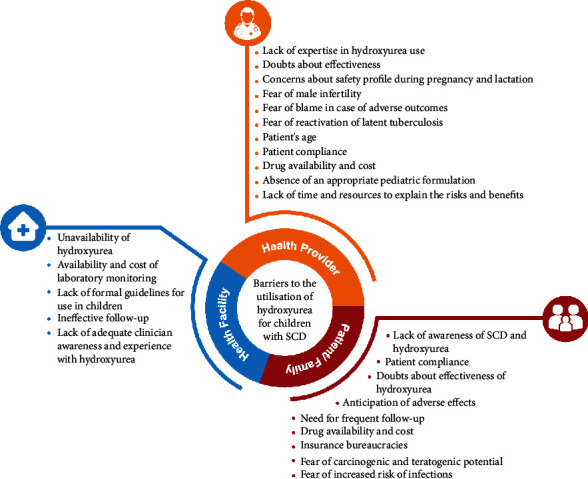
Barriers to utilization of hydroxyurea for children with SCD.

Hydroxyurea (hydroxycarbamide), a WHO essential medicine [130], is a safe, effective [131], and approved disease-modifying therapy for oral use in children with SCD in sub-Saharan Africa [132]. It has been extensively studied and prescribed in developed countries [131, 133] and yet underutilized in high-SCD-burden countries [134, 135]. In Nigeria, for instance, less than 1 percent of an estimated 1.2 million SCD population use HU [49]. Barriers to HU use range from health system to health providers, patients, and their caregivers (Figure 3) [21, 25, 136–138]. Unless HU is made widely available and multidimensional barriers to its use are addressed, families and patients will continue to experience poor quality of life, high medical costs, increased time out of school due to recurrent and prolonged hospitalization, lost family income, and reduced life expectancy [139].

Although HU leads to increased HbF levels, the actual mode of action is yet to be understood. HU is a ribonucleotide reductase inhibitor and is believed to have multiple mechanisms of action such as stimulation of NO production which has an effect on the vascular endothelium contributing to local vasodilation; decreased expression of adhesion molecules on erythrocytes, white blood cells, and vascular endothelium; reduced hemolysis through improved erythrocyte hydration, and macrocytosis [44, 133, 140]. Just like the baseline HbF level, response to HU therapy also varies among individuals, with only approximately two-thirds of patients with SCD responding to HU [141]. For instance, individuals with a baseline HbF level between 5% and 10% can have a 2- to 3-fold increase; whereas those with very low baseline HbF can have 10-fold increase on HU [142], implying that some genetic elements in the *β*-globin cluster predict a response to HU [143]. Ware and associates [144] documented that HbF response is affected by baseline HbF percentage, baseline HbF level, maximum tolerated dose, and treatment adherence.

#### 10.5.1. Indications for Hydroxyurea in SCD

Hydroxyurea was first synthesized by Dressler and Stein in 1869, in Germany. It was in the 1960s that its activity against myeloproliferative disorders was discovered [36, 145]. Use of HU for adults with SCD was approved by the United States Food and Drug Administration (FDA) in 1998, and subsequently for children in 2017 [37]. Hydroxyurea is recommended for patients with HbSS and HbS*β*^0^ thalassemia genotypes but can be considered for other genotypes on an individual basis [37, 127, 146]. It should be noted that indications vary among countries. Consensus guidelines for developed countries such as the United States recommend HU for all patients aged 9 months and above with SCA regardless of clinical severity [83]. British guidelines also recommend HU for infants with HbSS and HbS*β*^0^thal aged 9-42 months of life regardless of clinical severity. Children aged >42 months, adolescents, and adults should receive HU if they experience ≥3 moderate to severe VOC in a year, sickle cell pain that interferes with daily activities and quality of life, and a history of severe and/or recurrent ACS [127]. Countries in SSA, on the other hand, consider specific clinical criteria. For instance, guidelines of high-SCD-burden countries such as Nigeria [49] and Uganda [125] recommend HU in children and adults with abnormal TCD > 200 cm/s, stroke, ACS, > 5 crises per year, 3-4 crises per year, and either a steady-state neutrophil count > 10 x 10^9^ /L or platelet count > 500 x 10^9^/L. Tanzania on the other hand also recommends HU initiation for all children aged at least 9 months with confirmed SCD [105]. Other indications include recurrent priapism and patients with chronic kidney disease who are taking erythropoietin [72, 105]. It is unfortunate that in many instances, HU is started after a child has developed life-changing complications such as stroke.

#### 10.5.2. Laboratory and Clinical Benefits of Hydroxyurea in SCD

The earliest clinical trials on the effects of HU on children and adults with SCD were conducted in the early 90s [147, 148]. The beneficial effects of HU manifest after several months of therapy [147]. Hydroxyurea leads to a sustained increase in HbF levels and MCV and reduced WBC, reticulocyte, and platelet counts, and improves erythrocyte deformability and rheology, resulting in reduced vaso-occlusion and increased blood flow [83, 148, 149]. Sickling is inhibited by HbF, which interferes with the polymerization of HbS, because HbF lacks *β*-globin chains [32]. Individuals with SCD who have high HbF levels have delayed manifestation of symptoms, less severe disease [135], and lower risk of early death [150]. HU also results in increased total hemoglobin levels, reduced vaso-occlusive pain episodes and dactylitis, reduced need for blood transfusion, reduced hospitalizations and duration of hospitalization, decrease in glomerular hyperfiltration, and death [132, 146, 149, 151]. The Hydroxyurea Safety and Organ Safety (HUSOFT) extension trial [149] revealed a significant decrease in the occurrence of acute chest syndrome. Following four years of HU therapy for children with HbSS and and HbS*β*°, 7.5 acute chest syndrome events per 100 person years occurred among patients compared to 24.5 events per 100 person years among controls (*p* = 0.001). The TWiTCH study [152] provided insight that hydroxyurea therapy after at least one year of regular blood transfusion can prevent primary stroke in children with abnormal TCD velocities and without severe vasculopathy as defined by magnetic resonance angiography.

#### 10.5.3. Adverse Effects of Hydroxyurea

Hydroxyurea causes decreased white blood cell, reticulocyte, and platelet counts [146, 148, 149, 153]. Fortunately, severe toxicity due to hydroxyurea is rare [154], and these laboratory changes are usually transient and reversible when HU is temporarily stopped [153]. Some children taking HU may experience nausea, diarrhea, and headache, which are often mild [153]. Hydroxyurea is not associated with growth failure [153], and its use in African children is not associated with an increased risk of malaria or bacterial infections [132]. It is not unusual for clinicians and families to express concerns about the potential for HU to cause malignancy, male infertility, and leg ulcers. There is insufficient or weak evidence that HU is associated with these outcomes [83, 131, 155]. Notably, patients with SCD can spontaneously develop malignancies without HU therapy [156]. Case reports show that children [157] and adults [158] taking HU may develop skin and nail changes in patterns ranging from longitudinal pigmented bands, hyperpigmented nails (melanonychia), palmar creases, and macules. These changes may be observed within 6 to 12 weeks after initiation of HU therapy and at relatively lower doses of HU. They are non-pruritic, painless, and may disappear spontaneously without discontinuation of therapy [157]. Hydroxyurea is also not associated with hepatic and renal dysfunction [133]. More is yet to be learned about the long-term effects of HU in children with SCD, given that HU has only been used for a few decades in this population.

#### 10.5.4. Contraindications for Hydroxyurea in SCD

HU should not be administered to pregnant and breastfeeding women, and thus, pregnancy testing should be done for patients in child-bearing age prior to initiation of HU [83]. Women and men taking HU should employ methods of contraception, and when pregnancy is desired, they should discontinue HU at least 3 months before conception [72, 83, 126]. Patients with liver disease, renal disease, and low blood cell counts (absolute neutrophils <2,000/*μ*L, platelets <80,000/*μ*L, reticulocytes <100,000/*μ*L) should not receive HU [49, 83, 105]. According to some guidelines, HU therapy should not be started if Hb is less than 6g/dl [105]. As such, a complete blood count, reticulocyte count, liver, and renal function test panels should be performed prior to its initiation. ALT should be <2 times the upper limit of normal, and creatinine should be normal prior to starting HU [24]. When possible, HbF levels should be determined using tests such as hemoglobin electrophoresis or HPLC, although elevated HbF level should not deter the initiation of HU [83, 105].

#### 10.5.5. Prescription, Administration, and Monitoring of Hydroxyurea Therapy in SCD

Substantial differences in prescription practices exist. It is recommended that clinicians follow guidelines to optimize care and benefits [83]. As a prerequisite, clinicians, patients, and/or family members should hold a discussion about the indications, benefits, and adverse effects of HU, and they should be in a position to be regularly monitored [24].

HU is available as a liquid, capsule, and tablet [159]. Because HU is mostly available as 500mg and/or 250mg capsules in our setting and liquid formulations are not available on a commercial scale [72, 133], open the capsule and dissolve the contents in liquids or foods for infants and younger children [154]. A liquid formulation of HU with a concentration of 100 mg/mL can also be prepared and can be stable for more than 90 days at room temperature [160, 161]. In some countries, 100mg, 200mg, 300mg, and 400mg capsules of HU are commercially available, which facilitates accurate weight-based dosing [133, 161, 162].

An initial dose of 15 to 20mg/kg/day is prescribed, increased by 2.5 to 5mg/kg/day (maximum daily dose of 35mg/kg) every 8 weeks until a satisfactory clinical response is observed or until a maximum tolerable dose (the dose at which thrombocytopenia, neutropenia, or reticulocytopenia develops), whichever comes first [49, 65, 83]. In situations where only 500 capsules are available, clinicians are advised to calculate the weekly dose of HU in terms of the number of capsules needed per week and distribute the capsules across the week. For example, a 10kg patient initiated on a dose of 20mg/kg/day requires 200mg of HU daily and 1400mg per week. This patient receives three [3] 500mg capsules of HU every week and can therefore take 1 capsule on Monday, Wednesday, and Friday [105].

Perform a complete blood count (CBC) with WBC differential and reticulocyte count 4 weeks after initiation of HU, and 4 weeks after each dose escalation, with the goal of maintaining a neutrophil count of ≥2,000/*μ*L and a platelet count of ≥80,000/*μ*L [72, 83, 127]. Comparison should always be done between previous and current laboratory values [133]. If neutropenia and thrombocytopenia develop, stop HU therapy and repeat CBC with WBC differential every 1 to 2 weeks. Once blood counts have recovered, restart HU at a dose 5 mg/kg/day lower than that given before the onset of cytopenias [83]. Perform a CBC and reticulocyte count every 2-3 months after determining a stable dose of HU [83, 127]. Patients on HU should have their HbF levels monitored, as well as for adverse effects [49]. However, a failed increase in HbF levels does not indicate a need for discontinuation of HU therapy [83]. Patients should be advised to continue HU during illness or hospitalization except if neutropenic or bleeding with thrombocytopenia and should not double up doses if a dose is skipped [83, 127].

A trial of a minimum of 6 months on the maximum tolerated dose of HU should be given before consideration for discontinuation, because the optimal clinical and laboratory response to HU may take up to 6 to 12 months. Poor response mainly occurs due to failure of dose escalation or lack of adherence, and to a lesser extent due to frank failure to respond to therapy. In case poor adherence is identified, repeated discussions should be held with families and patients about the benefits of HU therapy, in addition to recognition and rectification of the reasons for poor adherence, because unnecessary dose escalation may result in toxicity and continued nonadherence. Nonresponse to HU is rare in children [133] and should be based on clinical criteria such as failure to reduce the frequency and severity of painful episodes or ACS, rather than laboratory criteria. Long-term HU therapy is indicated if a clinical response is documented, but adherence should be emphasized [83, 127]. A sickle cell expert should be consulted for patients who do not demonstrate a clinical response despite appropriate doses and duration of HU [83].

### 10.6. L-glutamine

Glutamine is an essential amino acid and precursor for the synthesis of glutathione, nicotinamide adenine dinucleotide (NAD), arginine, and nitric acid, which protect erythrocytes from oxidative damage and indirectly maintain vascular tone [33, 163]. L-glutamine (Endari®) was approved by the U.S. FDA in 2017 [164] for use in children aged 5 years and above, 20 years after approval of HU. This approval did not come without contention, given the lack of long-term safety data and the fact that studies have yielded conflicting clinical benefits of glutamine as an antioxidant in many situations [163]. As such, researchers call for more consolidating therapeutic trials to determine the impact of L-glutamine on SCD-associated morbidity and mortality [163]. Data from a phase II randomized, double-blind, placebo-controlled, parallel-group study [165] showed a significant reduction in acute vaso-occlusive pain and hospitalization among patients with HbSS or HbS*β*°-thalassemia after 6 months of therapy with L-glutamine. In a subsequent phase-3 multicenter double-blind RCT with a 48-week treatment and follow-up period [166], patients aged 5-58 years with HbSS or HbS*β*°-thalassemia who received L-glutamine (with or without HU) experienced a 25% reduction in acute pain episodes and 33% reduction in hospitalization respectively, compared to placebo (with or without HU). No significant differences were observed with regard to changes in Hb levels, hematocrit, or reticulocyte count, but patients in the L-glutamine arm were more likely to report nausea, chest pain (not of cardiac origin), fatigue, and musculoskeletal pain than the placebo group [166]. Data from this trial was the basis for FDA approval of L-glutamine. A single-center phase-4 trial (NCT04684381) to determine the pharmacokinetic characteristics and safety of L-glutamine in SCD is ongoing and is expected to be completed in 2022 [167]. L-glutamine is an amino acid that is available as an orally administered powder and capsule for oral suspension or mixing with food. L-glutamine therapy is given twice daily and does not require any monitoring [163, 168]. Major drawbacks include the cost, taste, and lack of biomarkers of response to L-glutamine [36, 163, 169]. Adverse effects of L-glutamine include nausea, fatigue, musculoskeletal pain, and chest pain of noncardiac origin [166].

### 10.7. Crizanlizumab

Crizanlizumab (Adakveo®) is a humanized monoclonal antibody that binds to P-selectin on endothelial cells and platelets and blocks its interaction with P-selectin glycoprotein ligand-1 (PSGL-1), thus preventing cell-to-cell interactions that are involved in the pathogenesis of vaso-occlusion in the microvasculature [170]. Evidence regarding the effect of crizanlizumab for the prevention of vaso-occlusive pain in SCD is encouraging. A double-blind, randomized, placebo-controlled, phase 2 trial involving 198 SCD patients, majority of black race (91.9%), aged 16 to 65 years, was conducted at 60 study sites located in the United States, Brazil, and Jamaica. Patients who had 2 to 10 vaso-occlusive pain crises during the 12 months prior to recruitment were eligible for randomization to high-dose crizanlizumab (5.0 mg/kg), low-dose crizanlizumab (2.5 mg/kg), and placebo groups, with 67, 66, and 65 participants assigned to these categories respectively. Crizanlizumab and placebo were administered intravenously 14 times over a 52-week period, and 129 participants completed the trial. A total of 69 (34.8%) participants exited the study early, 24 in the high-dose crizanlizumab group, 21 in the low-dose crizanlizumab group, and 24 in the placebo group. Patients on hydroxyurea therapy were required to have received it for not less than 6 months, should have been on a stable dose for at least 3 months prior to enrollment, and were not allowed to have any dose alteration during the 52-week treatment phase of the study except for safety reasons. In each group, 25 patients did not receive HU. The median rate of crises per year was 1.63 among patients who received high-dose crizanlizumab, and 2.98 among those who received placebo (*P* = 0.01). Patients who received high-dose crizanlizumab had a longer median time to the first crisis compared with placebo (4.07 versus 1.38 months, *P* = 0.001). Similar findings were reported regarding the median time to the second crisis (10.32 versus 5.09 months, *P* = 0.02). High-dose crizanlizumab resulted in a 62.9% lower median rate of uncomplicated crises per year compared with placebo (1.08 versus 2.91, *P* = 0.02). Notably, patients in the crizanlizumab group were less likely to develop events [170]. It is no wonder that in 2019, intravenously administered crizanlizumab received expedited FDA approval as the first targeted therapy for the prevention of VOP in SCD. Crizanlizumab may cause arthralgia, diarrhea, pruritus, vomiting, and chest pain [170].

A phase 2, multicenter open-label trial (NCT03474965) of 100 children aged 6 months to 17 years with any SCD genotype is ongoing [171]. The purpose is to establish the ideal dosing and safety of intravenously administered crizanlizumab in children with or without hydroxyurea.

### 10.8. Voxelotor

Voxelotor (GBT440, Oxbryta®) is a sickle hemoglobin polymerization inhibitor that works by increasing the oxygen affinity of hemoglobin [172]. It is administered as once daily oral tablets or oral suspension. In November 2019, the FDA granted accelerated approval of voxelotor for children and adults aged 12 years and above [173], and later for children aged 4 to 11 years in December 2021 [174]. Marketing authorization of voxelotor for the treatment of SCD in children and adults aged ≥12 years was granted in the European Union and United Arab Emirates [175]. These decisions followed convincing evidence from the HOPE trial [176], a phase 3, double-blind, randomized, placebo-controlled trial conducted at 60 institutions in 12 countries. Participants were patients with confirmed SCD aged 12 to 65 years whose hemoglobin level was between 5.5 and 10.5 g/dl during screening, and had experienced 1 to 10 vaso-occlusive crises in the past year. In addition, participants who were receiving hydroxyurea were eligible if they were on a stable dose for at least 3 months. Of the 274 participants, 90 were assigned to the 1500mg voxelotor arm, 92 to the 900mg voxelotor arm, and 92 to the placebo arm. At week 24 of follow-up, a hemoglobin rise of more than 1.0 g/dl above baseline was observed in 51% (95% CI: 41-61) of the 1500mg voxelotor arm compared to 7% (95% CI: 1-12) in the placebo arm (*P* < 0.001). One-third (33%, 95% CI, 23-42) of the 900mg voxelotor arm had a significant rise in Hb level at week 24 of follow-up. Hemoglobin levels of at least 10 g/dl at week 24 were observed in 41% of the participants in the 1500mg voxelotor arm, 20% in the 900mg voxelotor arm, and 9% in the placebo arm. The mean rise of Hb was consistent across the voxelotor group regardless of concurrent HU use or baseline Hb. In addition, the incidence of acute anemia episodes (decrease in the Hb level of >2.0 g/dl from baseline during the trial) was lower in the voxelotor arms than in the placebo arm. Participants in the 1500mg voxelotor arm had a significantly higher decrease in indirect bilirubin levels compared to those in the placebo arm, with a mean change of −29.1% and −3.2% respectively (*P* < 0.001). Similar findings were demonstrated in the phase 2a HOPE KIDS-I trial (NCT02850406) involving 45 children aged 4 to 11 years [177]. Just like L-glutamine and crizanlizumab, voxelotor has not yet been proven to prevent, delay, or improve organ-specific complications (e.g., renal disease, PAH), and improve quality of life or survival [45]. How these drugs can be combined to yield better outcomes is a story for the future.

Currently, a prospective observational study (NCT04930445) of 1000 participants at 45 study sites in the United States is ongoing and is projected to be completed in 2028 [178]. The study intends to evaluate the effect of Oxbryta in children and adults with SCD. In addition, a phase 3 randomized, double-blind placebo-controlled trial dubbed HOPE Kids 2 trial (NCT0421084) involving 224 children aged 2 to 14 years with SCD is underway, with the primary objective of evaluating the effect of voxelotor on TCD measurements [179]. The study is conducted across 43 sites located in the U.S, Europe (United Kingdom, France, Italy), Africa (Nigeria, Ghana, Egypt), and Asia (Saudi Arabia, Oman), and is projected to be completed in 2026.

### 10.9. Hematopoietic Stem Cell Transplant

The first success story of hematopoietic stem cell transplant (HSCT) was published in 1984 [180]. An 8-year-old girl with SCD was cured of acute myeloblastic leukemia after HSCT from an HLA-matched sister with HbAS. The patient was also cured of her SCD complications, showing that SCD can be cured without necessarily reversing to the normal hematological genotype (HbAA).

HSCT is now a well-established cure for SCD that is usually considered for patients with stroke, acute chest syndrome, and recurrent vaso-occlusive pain [181]. Stem cells are usually harvested from the bone marrow, peripheral blood, or cord blood [181]. Although less than 14% of individuals with SCA have HLA-matched siblings as potential donors [46], available data shows that HLA-haploidentical (half-matched) stem cell transplant is a viable alternative, with patients having a 91% overall survival and low transplantation-related toxicity [182], comparable with 92.9% (95% confidence interval: 91.1%-94.6%) overall survival among HLA-matched sibling HSCT [181]. This has been made possible by improvements in conditioning regimens, robust pre- and post-transplantation T-cell depletion, and improvement in supportive care [182].

Wider use of HSCT is limited by limited geographic expertise, availability of human leucocyte antigen- (HLA-) matched donors, and complicated by graft-versus-host disease (GVHD) and death, mostly attributed to infection [84, 181]. Many tertiary facilities in SSA lack infrastructure and funding to perform HSCT. Access to HSCT is limited to 6 of 54 African countries (South Africa, Nigeria, Morocco, Algeria, Tunisia, and Egypt) [183], and patients often seek HSCT in India, Canada, and U.S [184]. Nonetheless, high-burden countries such as Tanzania have accelerated progress in establishing HSCT capabilities through training health professionals both locally and through exchange programs with developed countries; research; establishment of partnerships with community and international organizations and experts in setting up and conducting HSCT; and mobilization of funds [184, 185].

## 11. Other Potential and Futuristic Therapies

### 11.1. Pipeline Agents

Several agents that target HbS polymerization (e.g., Mitapivat, decitabine, Senicapoc), pro-adhesive molecules/selectins (e.g., Rivipansel), and inflammation (e.g., Canakinumab, Regadenoson), among others [46] are either still under study or have been shown to ameliorate SCD severity. Perhaps, some of these agents will translate into SCD care and provide additional benefits.

### 11.2. Omega-3 Fatty Acids

Dietary omega-3 (*ω*-3 or n-3) fatty acids, principally docosahexaenoic acid (DHA), and eicosapentaenoic acid (EPA) are thought to have antioxidant, antithrombotic, and anti-inflammatory properties, and thus likely to modulate key pathophysiologic mechanisms in SCD [186]. These polyunsaturated fatty acids are found in oily fish and fish oil supplements and are substrates for the synthesis of mediators with anti-inflammatory and inflammation-resolving properties called resolvins, protectins, and eicosanoids [187, 188]. Erythrocyte membranes and plasma of patients with SCD have a high ratio of proinflammatory arachidonic acid (ARA) to DHA and EPA (high *ω*-6/*ω*-3 ratio) compared to controls, a finding which correlates with high sensitive C-reactive protein levels [189, 190]. Arachidonic acid is an *ω*-6 fatty acid that acts as a substrate for the production of eicosanoids that otherwise have inflammatory properties [187, 188].

Few studies have been conducted to establish the role of *ω*-3 fatty acids in ameliorating SCD complications. A small trial of adults (*n*=10) published nearly 20 years ago [191] showed that patients with SCD supplemented with dietary *ω*-3 fatty acids for 1 year experienced a decrease in the frequency of pain episodes requiring hospital care from 7.8 events during the previous year to 3.8 events per year (*P* < 0.01). A single-center randomized, placebo-controlled, double-blind trial [192] conducted in South Sudan involving 140 patients aged 2 to 24 years with SCA found that *ω*-3 fatty acids reduce the median rate of vaso-occlusive pain episodes from 4.6 to 2.7 per year (*P* < 0.01) and of pain requiring hospitalization from 1 to 0 per year (*P* < 0.0001). The annual frequency of severe anemia was also reduced from 16.4% to 3.2% (*P* < 0.05), whereas that of transfusion reduced from 16.4% to 4.5% (*P* < 0.03). Although this data provides insight regarding the potential benefits of *ω*-3 fatty acids in SCD, there is a need for a stronger body of evidence through a carefully planned and larger multicenter trial.

### 11.3. Gene Therapy

Gene therapy is a relatively new and rapidly developing science with both curative and disease-modifying potential for SCD. It was born out of an improved understanding of the molecular pathways that regulate erythropoiesis and globin switching in mammals, and improved gene transfer tools. Gene therapy broadly takes a variety of approaches including addition of a helpful gene, often using a lentiviral vector, gene knockdown (e.g., BCL11A silencing), direct globin gene editing to rectify the mutation (changing the HbS-encoding gene to HbA-encoding gene), and gene editing of elements that regulate globin, to at least partially reverse the normal hemoglobin switching from HbF to HbA (e.g., by targeting the BCL11A gene) [193, 194]. Recently published pilot studies have shown that single treatment using gene therapy techniques results in sustained production of HbA in most RBCs and increased total Hb and HbF levels, reduces hemolysis, and completely resolves severe vaso-occlusive events [195, 196], whereas variable findings have been documented in other studies [197, 198]. Several other pilot studies are underway to primarily assess the feasibility, safety, and efficacy of gene therapy in patients with severe SCD [198–200].

A recent trial of two patients (19 year-old with transfusion dependent thalassemia and 33 year-old with HbSS and a single *α*-globin deletion) showed that infusion of autologous CRISPR-Cas9–edited CD34+ hematopoietic stem and progenitor cells resulted in an early, significant, and sustained increase in total Hb and HbF levels, reduction in intracellular HbS levels, elimination of transfusion requirement, and acute vaso-occlusive pain episodes [201]. Sepsis and pneumonia were reported to occur in the presence of neutropenia, in addition to cholelithiasis, veno-occlusive liver disease with sinusoidal obstruction syndrome, and abdominal pain [201]. This revolutionary precision-based technology is still in experimental stages and will not be available anytime soon in low- and middle-income countries.

## 12. Adolescent to Adult Care Transition

**Figure 4:**
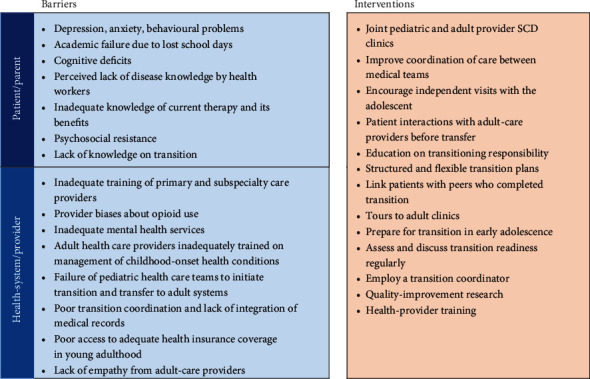
Barriers to adolescent-to-adult care transition and proposed interventions.

Programs that support the transition and integration of adolescents with SCD into adult-centered care are crucial, given that the life span of individuals with SCD continues to increase. Transition of adolescents into adult-centered care for SCD usually occurs at ≥18 years, but preparation for readiness to transition should begin a few years earlier [202, 203]. Patients aged 18 to 25 years can be reviewed by the pediatric and adult care hematologist and progressively bridged to only adult care by 26 years. However, it has been observed that adolescents and young adults with SCD experience increasing severity of complications, hospitalization, and mortality, particularly between 15 and 34 years, a period during which transition occurs [202, 204]. Furthermore, adolescents and young adults experience inadequate care in the emergency room and prefer their usual care physician, which compromises transition outcomes [205]. Ineffective transition is attributed to several patient, parent, health provider, and health system barriers. These barriers and potential interventions are summarized in Figure 4 [203, 205–207]. Perhaps, adult care providers should foster good relationships with patients, develop expertise to coordinate, and maintain optimal quality of care during and after transition, as well as develop tools to monitor the outcomes of transition.

For a healthcare transition program to be effective, it should be well structured and systematic. More precisely, a transition program comprises of six core elements, namely, (1) developing of a transition policy, (2) tracking and monitoring progress, (3) assessing transition readiness, (4) planning for adult care, (5) transfer to adult care, and (6) integrating into adult care [208]. While transition programs have been prioritized in high-income countries where individuals with SCD live for an average of 60 years [207], many countries and hospitals in SSA lack structured transition programs, and adult hematologists and nurses experienced in child-onset chronic illnesses are exceedingly rare. As such, individuals who survive into adulthood continue to attend pediatric SCD clinics and inpatient care provided by nurses, medical clinical officers, and general practitioners without an extensive understanding of the complexity of SCD complications in adults. There is no doubt that pragmatic trials are urgently needed to establish feasible approaches that facilitate readiness for a smooth transition to adult care in high-SCD-burden countries in SSA [206], and yet still maintain good quality of care and outcomes of adolescents and adults with SCD. Although this may be a highly complex matter, given the numerous health system challenges, we believe that continued research and patient/family inclusive quality improvement initiatives and development of quality indicators for transition readiness and successful transition [209] will be key in fostering success. However, we need to first optimize the collaboration between pediatric and adult care providers including medical doctors (general practitioners and specialists), clinical officers, and nurses. Besides, the concept of care transition of patients with chronic life-debilitating illnesses should be embedded in the curricula of undergraduate and postgraduate medical and nursing training institutions in SSA.

## 13. Prevention of SCD

Key approaches to the prevention of SCD include universal newborn screening, premarital and preconceptual genetic counselling and testing. Achieving this requires extensive community sensitization, availability, and accessibility to free or subsidized SCD screening services, which requires the involvement of key stakeholders such as religious, cultural, and political leaders, and village health teams [24].

## 14. Conclusion

Sickle cell disease is one of the most common monogenic diseases worldwide and is highly prevalent in SSA. It had been largely neglected as a global health priority until 2006 when the World Health Organization recognized it as a global health priority. More than 500 children die every day because of delayed diagnosis and lack of comprehensive care for SCD [15]. Implementation of newborn screening in sub-Saharan Africa is still in infancy, and yet, early diagnosis facilitates early enrolment into comprehensive care programs and family health education, hence reducing the morbidity and mortality associated with SCD. There is no doubt that this can be widely achieved through the use of POCTs, which are considerably cost-effective and accurate options for early screening of SCD in newborns. Their use should be integrated into existing preventive care programs such as immunization and HIV early infant diagnosis in high-burden countries in SSA [15]. Additionally, there is an urgent need to implement the widescale use of hydroxyurea in combination with other approved disease-modifying therapies (L-glutamine, crizanlizumab, voxelotor) for children with SCD in SSA. Since curative therapies (HSCT and gene therapy) are distant options for the majority of Africa's children with SCD, countries should improve the access and quality of comprehensive care. To further enhance the survival and quality of life of patients with SCD, countries in SSA should invest in health systems research to streamline the transition from adolescent-to-adult care.

## Figures and Tables

**Table 1:** Clinical presentation and complications of SCD.

System/organ	Complications
Neurological	Stroke (hemorrhagic or ischemic)
Eyes	Retinopathy
Respiratory	Acute chest syndrome, asthma
Cardiovascular	Cardiomyopathy, left ventricular hypertrophy, pulmonary artery hypertension (PAH), venous thromboembolism
Spleen	Acute splenic sequestration, chronic splenomegaly
Hepato-biliary	Sickle hepatopathy (hepatic sequestration, viral hepatitis, sickle cell intrahepatic cholestasis, cholelithiasis), transfusion iron overload
Renal	Proteinuria, painless hematuria, hyposthenuria, renal impairment/failure
Genital	Priapism
Bones and joints	Acute vaso-occlusive pain crisis, chronic pain, avascular necrosis, aplastic crisis, multifocal osteomyelitis, septic arthritis, fronto-occipital bossing, gnathopathy
Skin	Chronic leg ulcers
Other	Infections, girdle/mesenteric crisis, delayed growth and puberty, jaundice, pallor, depression, anxiety, and poor academic performance

References: [32, 34, 39, 46, 48, 49, 80, 83].

**Table 2:** Drugs used in sickle cell disease.

Drug	Recommended dose	Frequency	When to stop	Comments
Phenoxymethyl penicillin (Pen-V)	<1 year: 62.5mg	Oral, twice daily, beginning at 3 months of life	5 years, unless child had splenectomy or invasive pneumococcal infection	Prophylaxis against encapsulated bacteria
	1-3 years: 125 mg			
	≥3 years: 250 mg			
Erythromycin	Same dose as Pen-V			Macrolides for penicillin allergic patients

Sulfadoxine-pyrimethamine (e.g., Fansidar)	<2 years: ½ tablet	Oral, once every month	Life-time	Prophylaxis against malaria Begin from age >2 months
	2 to 5 years: 1 tablet			
	>5 years: dose based on weight			
Proguanil	<1 year: 25mg	Oral, once daily		Prophylaxis against malaria. Based on country's national guidelines Encourage other malaria prevention strategies
	1-3 years: 50mg			
	3-6 years: 50-100mg			
	>6 years: 100-200mg			

Folic acid	<1 year: 62.5 – 2.5 mg	Oral, once daily	Life-time	All children aged <3 years may receive 2.5mg daily
	1-3 years: 2.5 mg			
	≥3 years: 5 mg			

Hydroxyurea	15-20 mg/kg/day initial dose (max=35mg/kg/day)	Oral, once daily	See section 10.5	Escalate starting dose by 2.5 to 5mg/kg every 8 weeks until clinical response or hematological adverse effects

L-glutamineª	<30 kg: 5g (1 packet)	Oral, twice daily		Given to patients aged ≥5 years
	30-65 kg: 10g (2 packets			
	>65 kg: 15g (3 packets)			

Crizanlizumabª	5 mg/kg/dose	Intravenous, repeat dose after 2 weeks of the first, then every after 4 weeks		Given to patients aged ≥16 years

Voxelotor	1500 mg	Oral, once daily		Give to patients aged ≥12 years Improves anemia

ªCan be given with or without hydroxyurea. References: [10, 24, 33, 49, 83, 105, 113, 125].

**Table 3:** Screening/health monitoring of children and adolescents with sickle cell disease.

Investigation	Relevance	Timing	Interpretation of findings and interventions
TCD	Screening for risk of stroke	Begin at 2 years of age and continue until at least 16 years of age	Normal (all mean velocities <170 cm per sec). Continue TCD annually Conditional (mean velocity 170 to 199 cm per sec) ^¥^ Initiate hydroxyurea therapy Abnormal (mean velocity ≥200 cm per sec). Repeat TCD within 2 to 6 weeks. Start long-term transfusion therapy. If not possible, initiate HU therapy Inadequate (no information available on one or both middle cerebral arteries)
	Assesses blood velocity in the distal internal carotid, anterior or middle cerebral artery		

Ophthalmoscopy (Dilated retinal exam)	Ischemic retinopathy	Begin at 10 years, then every 1-2 years if normal	Refer patients with suspected retinopathy to a retinal specialist for possible laser photocoagulation therapy

Echocardiography	Screening for PAH		

Transcutaneous O2 saturation		Begin at 12 months, continue annually or more frequently based on clinical course	

Complete blood count with WBC differential and reticulocyte counts		Every 3 months beginning from 3 months of life, then every 6 months after 2 years of life	Frequency adjusted based on the patient's clinical state

Liver and renal function tests	Routine screening	Every 6 months	

HbF percentage		Every 6 months for children aged 6 to 24 months, then annually	

Spot urine testing	Microalbuminuria and proteinuria. Early markers of renal dysfunction	Begin by 10 years of life, and then annually if negative	If proteinuria (>300 mg per 24 hours), perform a first morning void urine albumin-creatinine ratio or 24-hour urine creatinine clearance. Consult/refer to nephrologist if abnormal ACEIs may be indicated

TCD: transcranial Doppler ultrasonography; ACEI: angiotensin-converting enzyme inhibitors. ^¥^Repeat TCD USS after 3 months, then every 4 months. References: [10, 24, 49, 83, 122, 126, 127].

**Table 4:** Diagnosis and treatment of acute complications of SCD.

Complication	Clinical presentation and evaluation	Treatment
Fever	May be a manifestation of an acute and sometimes life-threatening complication such as acute chest syndrome (ACS) or osteomyelitis. CBC with WBC differential, reticulocyte count, blood culture, and sensitivity. Perform urine culture and lumbar puncture for CSF analysis if urinary tract infection or meningitis is suspected respectively. Chest X-ray if signs of ACS	Immediate medical attention Temperature ≥39.5°C: admit to hospital for a 7-day course of IV antibiotics and close monitoring. Temperature ≥38.5°C: administer IV antibiotics with coverage against Streptococcus pneumoniae and gram-negative enteric organisms (e.g., ceftriaxone, 75-100 mg/kg), then subsequent outpatient care and follow-up within 24hr. Continue with an oral antibiotic is feasible in patients who do not appear ill or toxic looking. Subsequent antibiotic change should be based on culture and sensitivity results

Vaso-occlusive crises (VOC)	Manifests as sudden or gradual excruciating pain, most commonly in limbs, back, chest, and abdomen. Triggers include infections, stress, and cold exposure, among others Determine associated symptoms, location, and severity of pain Severity of pain is based on the patient report. Use a pain severity assessment scale such as the Wong-Baker facies	Treatment is individualized. Depends on severity of pain, patient or caregiver knowledge of predictably effective agents and doses, and previous adverse events. Initiate analgesics within 30 minutes of triage. ***Mild pain*:** non-opioid analgesics. Paracetamol 15mg/kg/dose 4 to 6hrly (maximum = 60mg/kg/day) +/- Ibuprofen 5-10mg/kg/dose 6-8 hrly OR diclofenac 1mg/kg PO/suppository 8hrly +/- adjuvants (i.e. anxiolytics, antidepressants) ***Moderate pain*:** non-opioid analgesics as above alternated with an opioid, e.g., codeine 0.5-1mg/kg every 3-4 h, dihydrocodeine 1mg/kg/dose 8hrly, oral oxycodone 0.15-0.20 mg/kg every 3-4 h, hydrocodone 0.15-0.20 mg/kg every 3-4 h, and tramadol IV 2 hr, +/- adjuvants Ketorolac 0.5 mg/kg/dose 6 hr (max=30mg/dose, 60mg/day). Avoid use with other NSAIDs. ***Severe pain:*** Oral morphine sulfate 0.2-0.4mg/kg/dose (max=20mg/dose) or diamorphine 0.1mg/kg/dose in IV infusion or IM/SC stat for immediate pain relief, then maintenance oral morphine 0.2-0.4mg/kg/dose 3-4 hourly. IV diclofenac 1mg/kg/dose 8 hourly after opioids Morphine IV or SC (0.05–0.15mg/kg over 10 minutes) 2-4hr or hydromorphone IV 0.015-0.020 mg/kg 3-4hr. In case of opioid-induced respiratory depression, give naloxone IV 10 *μ*g/kg (Max=8mg). Patient-controlled analgesia (PCA) may be provided Consider ketamine infusion at analgesic dose (0.1–0.3 mg/kg/hr, Max=1 mg/kg/hr) if pain is refractory to opioids When using morphine, administer laxatives (e.g., lactulose, senna) because it can cause constipation. Treat pruritus (itching) after opioid administration using oral antihistamines, and nausea/vomiting using antiemetics Avoid meperidine (risk of neurotoxicity – dysphoria, irritable mood, clonus, and seizures) unless it is the only effective opioid (0.75-1.0 mg/kg 3-4hr) Reassess patient after every 15-30 minutes to determine effectiveness of pain medication (+adverse events) and adjust accordingly Oral hydration. If unable to drink fluids, provide IV hydration at maintenance fluid rate Oxygen therapy if SPO2 <95% on room air Adjunctive nonpharmacological approaches (e.g., local warm application, massage, reassurance, and distraction through stories and play) Psychosocial support Rule out and treat malaria or bacterial infections (including osteomyelitis, septic arthritis) if confirmed, avascular necrosis, ACS Do not transfuse with blood unless other indication is present

Splenic sequestration crisis	Due to sudden progressive enlargement of the spleen caused by pooling of blood in the spleen. Quick drop in Hb level ≥2 g/dL below the baseline value. Can cause hypovolemic shock and death. Common in children <6 years with HbSS and some older children and adults with HbSC or HbS*β*-thal CBC and reticulocyte count (reticulocytosis, elevated circulating nucleated RBCs, anemia, and thrombocytopenia)	10ml/kg of packed RBCs or 15ml/kg of whole blood over 2-4hr to raise Hb to stable level, but not exceeding 8g/dl. Risk of hyperviscocity. Immediate IV fluid resuscitation if hypovolemia – normal saline 20ml/kg Monitor vitals Analgesics for pain Investigate and treat infections, malaria Monitor splenic size 12-24hr Splenectomy if ≥2 episodes occur, and in patient with chronic hypersplenism

Aplastic crisis	Acute acquired red cell aplasia caused by parvovirus B19 infection Presents with weakness and easy tiredness, fever, facial erythema, headache, severe anemia, and low reticulocytopenia (<2% of total RBC count)	Isolate patient – droplet precautions Transfuse with RBCs. Target to achieve the patient's steady-state Hb level

Acute chest syndrome	Caused by pulmonary infection, sequestration of RBCs in pulmonary vasculature, and fat embolism. May follow VOC or surgery Associated with high mortality Presents with cough, chest pain, difficulty in breathing, ± fever. Examination may be normal or reveal signs of respiratory distress, hypoxemia, wheezing, percussion dullness. Consider two or more of the above. New opacity on a chest X-ray Perform CBC, blood grouping and cross-matching, CRP, blood gas analysis if in respiratory failure	Hospital admission Oxygen therapy if low O2 saturation (target SPO2 >95%) Antibiotics: Third generation cephalosporin (e.g., ceftriaxone 80-100 mg/kg/day for 7-10 days) combined with an oral macrolide for Mycoplasma and Chlamydophila coverage: azithromycin 5-10mg/kg (max dose 500mg) once daily for 5 days or erythromycin 5-10 mg/kg/dose (max dose 500mg) 6 hourly for 7-10 days Bronchodilators (e.g., nebulized salbutamol 2.5 mg for children <5 years, 5mg for older children) Analgesics If Hb concentration is >1.0 g/dL below baseline, consider simple blood transfusion with 10mL/kg of packed RBCs or 20ml/kg of whole blood. Urgent exchange transfusion if rapid progression of ACS Optimal hydration (avoid pulmonary edema) Monitor vitals and for acute anemia Incentive spirometry every 2–4 hours while awake

Acute stroke	Ischemic and hemorrhagic stroke are mostly common in children and adults respectively Headache, vomiting, seizures, sensory/motor neurological deficits (paresis, hemiplegia, paraplegia, facial droop, aphasia), altered level of consciousness/coma MRI and MRA, acute brain infarct or hemorrhage CT, acute hemorrhage in the brain Exclude meningitis	Monitor vitals, maintain normal temperature Exchange blood transfusion within 4 hours if acute stroke is confirmed by neuroimaging Initiate long-term blood transfusion every 3-4 weeks and/or Hydroxyurea (if monthly transfusion unavailable) Physiotherapy in the long-term

Priapism	Presents as a sustained and painful erection unrelated to sexual stimulation. Lasts ≥4 hours (fulminant or major) or repeated painful erections lasting more than 30 minutes and up to 4-6 hours (stuttering) Erectile dysfunction and impotence can result from delay in diagnosis and treatment	At onset (<2hr): encourage extra oral fluids Oral or parenteral analgesia Attempt to urinate Warm birth Exercise (e.g., walking) Anxiolytics if anxious (e.g., lorazepam 0.05mg/kg/dose 8-12hr, Max= 2mg/dose) If >2hr, catheterize if unable to urinate, consult urologist or surgeon – to consider irrigation and aspiration of the corpus cavernosum and intracorporeal etilefrine or phenylephrine injection If no response to initial treatment, consider simple or exchange blood transfusion

Multisystem organ failure (MSOF)	Associated with VOC and characterized by respiratory, hepatic, and renal failure Unexpected and rapid deterioration, usually after several days of in-hospital treatment for severe VOC, at a time when pain is beginning to improve Fever, non-focal encephalopathy, ACS. Rapid decline in Hb and platelet count. Marked elevations in liver enzymes, total and direct bilirubin, blood coagulation screening tests. Elevated serum creatinine (± oliguria and hyperkalemia)	Rapid diagnosis and treatment Simple or exchange blood transfusion to Hb 10g/dL Supplemental oxygen and mechanical ventilation (if needed) Renal replacement therapy (e.g., hemodialysis) for acute renal failure (if needed)

Acute severe anemia	Hemoglobin <5g/dl or acute drop of Hb by >2g/dl from baseline/steady state or acutely symptomatic anemia Rule out malaria, bacterial infections, splenic or hepatic sequestration, and aplastic crisis	Immediately transfuse with packed RBCs 10ml/kg if symptomatic or Hb <5g/dl Transfuse to steady-state Hb if asymptomatic and Hb >5g/dl Treat patient according to other underlying diagnoses

CT: computerized tomography; MRA: magnetic resonance angiography; MRI: magnetic resonance imaging. References: [24, 49, 72, 83, 105, 106, 125, 126, 128, 129].

## Data Availability

All material supporting the conclusion of this review has been included within the article.

## Abbreviations

Hb: Hemoglobin
HPFH: Hereditary persistence of fetal hemoglobin
HU: Hydroxyurea
NBS: Newborn screening
POCT: Point-of-care test
RBC: Red blood cell
SSA: Sickle cell anemia
SCD: Sickle cell disease
SCT: Sickle cell trait
SSA: Sub-Saharan Africa.

## References

[1] Herrick J. B. (1910). Peculiar elongated and sickle-shaped red blood corpuscles in a case of severe anemia. *Archives Of Internal Medicine*.

[2] Savitt T. L., Goldberg M. F. (1989). Herrick’s 1910 case report of sickle cell anemia: the rest of the story. *JAMA*.

[3] Horton J. (1874). *The diseases of tropical climates and their treatment*.

[4] Ameh S. J., Tarfa F. D., Ebeshi B. U. (2012). Traditional herbal management of sickle cell anemia: lessons from Nigeria. *Anemia*.

[5] Nzewi E. (2001). Malevolent Ogbanje: recurrent reincarnation or sickle cell disease?. *Social Science & Medicine*.

[6] Semakula J. (2016). *How Buganda has battled sickle cells for 3-centuries*.

[7] World Health Organization (2010). Sickle cell disease: A strategy for the WHO African region [Internet]. https://www.afro.who.int/sites/default/files/2017-06/afr_rc60_8.pdf.

[8] United Nations (2009). Secretary-General’s message on sickle-cell disease [Internet]. https://www.un.org/sg/en/content/sg/statement/2009-06-19/secretary-generals-message-sickle-cell-anaemia.

[9] Ministry of Health Press statement ahead of World Sickle Cell Day [Internet]. https://www.health.go.ug/document/press-statement-ahead-of-world-sickle-cell-day/.

[10] Yawn B. P., John-Sowah J. (2015). Management of sickle cell disease: recommendations from the 2014 expert panel report. *American Family Physician*.

[11] Piel F. B., Tatem A. J., Huang Z., Gupta S., Williams T. N., Weatherall D. J. (2014). Global migration and the changing distribution of sickle haemoglobin: a quantitative study of temporal trends between 1960 and 2000. *The Lancet Global Health*.

[12] Rees D. C. (2022). Starting to understand the natural history of sickle cell disease in African countries. *The Hematologist*.

[13] Grosse S. D., Odame I., Atrash H. K., Amendah D. D., Piel F. B., Williams T. N. (2011). Sickle cell disease in Africa. *American Journal Of Preventive Medicine*.

[14] Piel F. B., Patil A. P., Howes R. E. (2013). Global epidemiology of sickle haemoglobin in neonates: a contemporary geostatistical model-based map and population estimates. *The Lancet*.

[15] Mcgann P. T. (2016). Time to invest in sickle cell anemia as a global health priority. *Pediatrics*.

[16] Nnodu O. E., Sopekan A., Nnebe-agumadu U. (2020). Implementing newborn screening for sickle cell disease as part of immunisation programmes in Nigeria: a feasibility study. *The Lancet Haematology*.

[17] Ohene-frempong K., Oduro J., Tetteh H., Nkrumah F. (2008). Screening newborns for sickle cell disease in Ghana. *Pediatrics*.

[18] Hernandez A. G., Kiyaga C., Howard T. A. (2021). Trends in sickle cell trait and disease screening in the Republic of Uganda, 2014 – 2019. *Tropical Medicine & International Health*.

[19] Oron A. P., Chao D. L., Ezeanolue E. E. (2020). Caring for Africa’s sickle cell children: will we rise to the challenge?. *BMC Medicine*.

[20] Simpson S. (2019). Sickle cell disease: a new era. *The Lancet Hematologist*.

[21] Aliyu Z. Y., Aliyu B., Mamman A. (2007). Hydroxyurea utilization in Nigeria, a lesson in public health. *Blood*.

[22] Quinn C. T., Rogers Z. R., McCavit T. L., Buchanan G. R. (2010). Improved survival of children and adolescents with sickle cell disease. *Blood*.

[23] Elmariah H., Garrett M. E., De Castro L. M. (2014). Factors associated with survival in a contemporary adult sickle cell disease cohort. *American Journal Of Hematology*.

[24] Ministry of Health (2020). *The National Guidelines for Management and Prevention of Sickle Cell Disease*.

[25] Kilonzi M., Mlyuka H. J., Felician F. F. (2021). Barriers and facilitators of use of Hydroxyurea among children with sickle cell disease: experiences of stakeholders in Tanzania. *Hemato*.

[26] Zounon O., Anani L., Latoundji S., Clay P., Mullet E. (2012). Misconceptions about sickle cell disease (SCD) among lay people in Benin. *Preventive Medicine*.

[27] Strunk C. (2021). *Dispelling common sickle cell disease myths*.

[28] Bharat D. (2021). *World sickle cell day: myths and facts associated with this inherited blood ailment*.

[29] Marengo-Rowe A. J. (2006). Structure-function relations of human hemoglobins. *Baylor University Medical Center Proceedings*.

[30] Schechter A. N. (2008). Hemoglobin research and the origins of molecular medicine. *Blood*.

[31] Schnog J. B., Duits A. J., Muskiet F. A. J., Cate H., Rojer R. A., Brandjes D. P. M. (2004). Sickle cell disease; a general overview. *Netherlands Journal Medicine*.

[32] Mccavit T. L. (2012). Sickle cell disease. *Pediatrics Review*.

[33] Brandow A. M., Liem R. I. (2022). Advances in the diagnosis and treatment of sickle cell disease. *Journal of Hematology & Oncology*.

[34] Kanter J., Kruse-jarres R. (2013). Management of sickle cell disease from childhood through adulthood. *Blood Reviews*.

[35] Streetly A., Sisodia R., Dick M., Latinovic R., Hounsell K., Dormandy E. (2018). Evaluation of newborn sickle cell screening programme in England: 2010–2016. *Archives Of Disease In Childhood*.

[36] Cisneros G. S., Thein S. L. (2021). Research in sickle cell disease: from bedside to bench to bedside. *Hemasphere*.

[37] American Society of Hematology (2021). Hydroxyurea for sickle cell disease. https://www.hematology.org/education/patients.

[38] Tubman V., Bennett C. M., Luo H., Chui D. H. K., Heeney M. M. (2007). Sickle cell disease caused by Hb S/Que´bec-CHORI: treatment with Hydroxyurea and response. *Pediatrics Blood Cancer*.

[39] Khamees I., Ata F., Choudry H., Soliman A. T., De S. V., Yassin M. A. (2021). Manifestations of HbSE sickle cell disease: a systematic review. *Journal of Translational Medicine*.

[40] Brown M. (2012). Managing the acutely ill adult with sickle cell disease. *British Journal of Nursing*.

[41] Wun T. (2001). The role of inflammation and leukocytes in the pathogenesis of sickle cell disease. *Hematology*.

[42] Ansari J., Gavins F. N. E. (2019). Ischemia-reperfusion injury in sickle cell disease: from basics to therapeutics. *The American Journal of Pathology*.

[43] Turhan A., Weiss L. A., Mohandas N., Coller B. S., Frenette P. S. (2002). Primary role for adherent leukocytes in sickle cell vascular occlusion: a new paradigm. *Proceedings of the National Academy of Sciences*.

[44] Johnson C., Telen M. J. (2008). Adhesion molecules and hydroxyurea in the pathophysiology of sickle cell disease. *Haematologica*.

[45] Telen M. J. (2020). Curative vs targeted therapy for SCD: does it make more sense to address the root cause than target downstream events?. *Blood Advances*.

[46] Williams T. N., Thein S. L. (2018). Sickle cell anemia and its phenotypes. *Annual Review Of Genomics And Human Genetics*.

[47] Schaer D. J., Vinchi F., Ingoglia G., Tolosano E., Buehler P. W. (2014). Haptoglobin, hemopexin, and related defense pathways basic science, clinical perspectives, and drug development. *Frontiers In Physiology*.

[48] Serjeant G. R. (2013). The natural history of sickle cell disease. *Cold Spring Harbor Perspectives in Medicine*.

[49] Federal Ministry of Health (2014). *National guideline for the control and management of sickle cell disease*.

[50] Shriner D., Rotimi C. N. (2018). Whole-genome-sequence-based haplotypes reveal single origin of the sickle allele during the Holocene Wet Phase. *The American Journal of Human Genetics*.

[51] Elderdery A. Y., Mills J., Mohamed B. A. (2012). Molecular analysis of the *β*-globin gene cluster haplotypes in a Sudanese population with sickle cell anaemia. *International Journal Of Laboratory Hematology*.

[52] Piel F. B., Patil A. P., Howes R. E. (2010). Global distribution of the sickle cell gene and geographical confirmation of the malaria hypothesis. *Nature Communications*.

[53] (2014). Reappraisal of known malaria resistance loci in a large multicenter study. *Nature Genetic*.

[54] Nelson D. A., Deuster P. A., Carter R., Hill O. T., Wolcott V. L., Kurina L. M. (2016). Sickle cell trait, rhabdomyolysis, and mortality among U.S. army soldiers. *New England Journal of Medicine*.

[55] Xu J. Z., Thein S. L. (2019). The carrier state for sickle cell disease is not completely harmless. *Haematologica*.

[56] Naik R. P., Smith-Whitley K., Hassell K. L. (2018). Clinical outcomes associated with sickle cell trait: a systematic review. *Annals Of Internal Medicine*.

[57] Lobitz S., Telfer P., Cela E. (2018). Newborn screening for sickle cell disease in Europe: recommendations from a Pan-European Consensus Conference. *British Journal Of Haematology*.

[58] El-haj N., Hoppe C. C. (2018). Newborn screening for SCD in the USA and Canada. *International Journal of Neonatal Screening*.

[59] Rahimy M. C., Gangbo A., Ahouignan G., Alihonou E. (2009). Newborn screening for sickle cell disease in the Republic of Benin. *Journal Of Clinical Pathology*.

[60] Nkya S., Mtei L., Soka D. (2019). Newborn screening for sickle cell disease: an innovative pilot program to improve child survival in Dar es Salaam. *Tanzania International Health*.

[61] Tshilolo L., Aissi L. M., Lukusa D. (2009). Neonatal screening for sickle cell anaemia in the Democratic Republic of the Congo: experience from a pioneer project on 31 204 newborns. *Journal Of Clinical Pathology*.

[62] Archer N. M., Inusa B., Makani J. (2022). Enablers and barriers to newborn screening for sickle cell disease in Africa: results from a qualitative study involving programmes in six countries. *BMJ Open*.

[63] Ndeezi G., Kiyaga C., Hernandez A. G. (2016). Burden of sickle cell trait and disease in the Uganda Sickle Surveillance Study (US3): a cross-sectional study. *The Lancet Global Health*.

[64] American Society of Hematology (2021). Consortium on Newborn Screening in Africa (CONSA) Highlights the Need for Newborn Screening of Sickle Cell Disease in Africa. https://www.hematology.org/newsroom/press-releases/2021/consa-highlights-the-need-for-newborn-screening-of-sickle-cell-disease-in-africa.

[65] de Montalembert M., Tshilolo L., Allali S. (2019). Sickle cell disease: a comprehensive program of care from birth. *Hematology 2014, the American Society of Hematology Education Program Book*.

[66] Makani J., Ofori-Acquah S. F., Nnodu O., Wonkam A., Ohene-Frempong K. (2013). Sickle cell disease: new opportunities and challenges in Africa. *The Scientific World Journal*.

[67] Nnodu O., Isa H., Nwegbu M. (2019). Hemo Type SC, a low-cost point-of-care testing device for sickle cell disease: promises and challenges. *Blood Cells, Molecules, and Diseases*.

[68] Steele C., Sinski A., Asibey J. (2019). Point-of-care screening for sickle-cell disease in low-resource settings: a point-of-care screening for sickle cell disease in low-resource settings: a multi-center evaluation of Hemo Type SC, a novel rapid test. *American Journal Of Hematology*.

[69] Oluwole E. O., Adeyemo T. A., Osanyin G. E., Odukoya O. O., Kanki P. J., Afolabi B. B. (2020). Feasibility and acceptability of early infant screening for sickle cell disease in Lagos, Nigeria — a pilot study. *PLoS One [Internet]*.

[70] Danho J. B. K., Atiméré Y. N., Koné D., Yéo D. D., Couitchéré L. (2021). Feasibility study of the “Hemo Type SC” test for the rapid screening of sickle cell disease in Côte D’Ivoire. *Advances Hematology*.

[71] Segbena A. Y., Guindo A., Buono R. (2018). Diagnostic accuracy in field conditions of the sickle SCAN ® rapid test for sickle cell disease among children and adults in two West African settings: the DREPATEST study. *BMC Hematology*.

[72] Ministry of Health (2020). *National guidelines for control and management of sickle cell disease in kenya*.

[73] Mvundura M., Kiyaga C., Kamya C., Lim J. M., Maiteki-Sebuguzi C., Coffey P. S. (2019). Cost for sickle cell disease screening using isoelectric focusing with dried blood spot samples and estimation of price thresholds for a point-of- care test in Uganda. *Journal Of Blood Medicine*.

[74] Okwi A. L., Byarugaba W., Ndugwa C. M., Parkes A., Ocaido M., Tumwine J. K. (2009). Knowledge gaps, attitude and beliefs of the communities about sickle cell disease in Eastern and Western Uganda. *East African Medical Journal*.

[75] Dennis-antwi J. A., Culley L., Hiles D. R., Dyson S. M. (2011). I can die today, I can die tomorrow’: lay perceptions of sickle cell disease in Kumasi, Ghana at a point of transition. *Ethnicity & Health*.

[76] Marsh V., Kombe F., Fitzpatrick R., Williams T. N., Parker M., Molyneux S. (2013). Consulting communities on feedback of genetic findings in international health research: sharing sickle cell disease and carrier information in coastal Kenya. *BMC Medical Ethics*.

[77] Steinberg M. H., Chui D. H. K., Dover G. J., Sebastiani P., Alsultan A. (2014). Fetal hemoglobin in sickle cell anemia: a glass half full?. *Blood*.

[78] Osafo-Kwaako A., Kimani K., Ilako D. (2011). Ocular manifestations of sickle cell disease at the korle-bu hospital, Accra. *Ghana European Journal of Ophthalmology*.

[79] Stevens M., Padwick M., Serjeant G. (1981). Observations on the natural history of dactylitis in homozygous sickle cell disease. *Clinical Pediatrics*.

[80] Oredugba F. A., Savage K. O. (2002). Anthropometric findings in Nigerian children with sickle cell disease. *Pediatric Dentistry*.

[81] Munube D., Katabira E., Ndeezi G. (2016). Prevalence of stroke in children admitted with sickle cell anaemia to Mulago Hospital. *BMC Neurology*.

[82] Uyoga S., Macharia A. W., Mochamah G. (2019). The epidemiology of sickle cell disease in children recruited in infancy in Kilifi, Kenya: a prospective cohort study. *The Lancet Global Health*.

[83] National Institute of Health (2014). *Evidence-based management of sickle cell disease*.

[84] American Society of Hematology (2022). Sickle cell research priorities. https://www.hematology.org/research/sickle-cell-disease-and-sickle-cell-trait/sickle-cell-research-priorities.

[85] Wonkam A., Chimusa E. R., Mnika K. (2020). Genetic modifiers of long-term survival in sickle cell anemia. *Clinical and Translational Medicine*.

[86] Steinberg M. H. (2005). Predicting clinical severity in sickle cell anaemia. *British Journal Of Haematology*.

[87] Weatherall M. W., Higgs D., Weatherall D., Serjeant G. R. (2005). Phenotype/genotype relationships in sickle cell disease: a pilot twin study. *Clinical & Laboratory Haematology*.

[88] Tewari S., Brousse V., Piel F. B., Menzel S., Rees D. C. (2015). Environmental determinants of severity in sickle cell disease. *Haematologica*.

[89] Sadreameli S. C., Kopp B. T., Creary S. E., Eakin M. N., Mcgrath-morrow S., Strouse J. J. (2016). Secondhand smoke is an important modifiable risk factor in sickle cell disease: a review of the current literature and areas for future research. *International Journal Of Environmental Research And Public Health*.

[90] West D. C., Romano P. S., Azari R., Rudominer A., Holman M., Sandhu S. (2003). Impact of environmental tobacco smoke on children with sickle cell disease. *Archives Of Pediatrics & Adolescent Medicine*.

[91] Tolu S. S., Reyes Gil M., Ogu U. O. (2019). High hemoglobin F in sickle cell disease: Waning protection with age. *Blood*.

[92] Khandros E., Huang P., Peslak S. A. (2020). Understanding heterogeneity of fetal hemoglobin induction through comparative analysis of F and A erythroblasts. *Blood*.

[93] Mouélé R., Pambou O., Feingold J., Galactéros F. (2000). *α*-Thalassemia in Bantu population from Congo-Brazzaville: its interaction with sickle cell anemia. *Human Heredity*.

[94] Rumaney M. B., Josiane V., Bitoungui N. (2014). The co-inheritance of alpha-thalassemia and sickle cell anemia is associated with better hematological indices and lower consultations rate in Cameroonian patients and could improve their survival. *PLoS One*.

[95] Embury S. H., Clark M. R., Monroy G., Mohandas N. (1984). Concurrent sickle cell anemia and alpha-thalassemia. Effect on pathological properties of sickle erythrocytes. *The Journal Of Clinical Investigation*.

[96] Powars D., Hiti A. (1993). Sickle cell anemia: *β* gene cluster haplotypes as genetic markers for severe disease expression. *American Journal of Diseases of Children*.

[97] Mears J. G., Lachman H. M., Labie D., Nagel R. L. (1983). Alpha-thalassemia is related to prolonged survival in sickle cell anemia. *Blood*.

[98] Nagel R., Fabry M., Pagnier J. (1985). Hematologically and genetically distinct forms of sickle cell anemia in Africa: the Senegal type and the Benin type. *New England Journal of Medicine*.

[99] Steinberg M. H., Lu Z. H., Barton F. B., Terrin M. L., Charache S., Dover G. J. (1997). Fetal hemoglobin in sickle cell anemia: determinants of response to hydroxyurea. *Blood*.

[100] Habara A. H., Shaikho E. M., Steinberg M. H. (2017). Fetal hemoglobin in sickle cell anemia: the Arab-Indian haplotype and new therapeutic agents. *American Journal Of Hematology*.

[101] Lee A., Thomas P., Cupidore L., Serjeant B., Serjeant G. (1995). Improved survival in homozygous sickle cell disease: lessons from a cohort study. *BMJ*.

[102] Rahimy M. C., Gangbo A., Ahouignan G. (2003). Effect of a comprehensive clinical care program on disease course in severely ill children with sickle cell anemia in a sub-Saharan African setting. *Blood*.

[103] Brown B. J., Madu A., Sangeda R. Z. (2021). Utilization of pneumococcal vaccine and penicillin prophylaxis in sickle cell disease in three African countries: assessment among healthcare providers in Sickle in Africa. *Hemoglobin*.

[104] Kambale-kombi P., Marini R., Alworong J. (2021). Management of sickle cell disease: current practices and challenges in a northeastern region of the Democratic Republic of the Congo. *Hematology*.

[105] United Republic of Tanzania (2020). *Ministry of Health, Community development, gender, elderly and C. Sickle cell disease clinical management guidelines*.

[106] Indiana Hemophilia, Thrombosis Center Sickle cell disease education: care of patients with sickle cell disease for primary care providers and emergency room personnel. https://www.in.gov/health/gnbs/files/SCD_Sickle_Cell_Disease_Education.pdf.

[107] Frimpong A., Thiam L. G., Arko-boham B., Dedea E., Owusu A., Adjei G. O. (2018). Safety and effectiveness of antimalarial therapy in sickle cell disease: a systematic review and network meta-analysis. *BMC Infectious Diseases*.

[108] Nakibuuka V., Ndeezi G., Nakiboneka D., Ndugwa C. M., Tumwine J. K. (2009). Presumptive treatment with sulphadoxine-pyrimethamine versus weekly chloroquine for malaria prophylaxis in children with sickle cell anaemia in Uganda: a randomized controlled trial. *Malaria Journal*.

[109] Dawam J., Madaki J., Gambazai A. (2016). Monthly Sulphadoxine-Pyrimethamine combination versus daily Proguanil for malaria chemoprophylaxis in sickle cell disease: a randomized controlled study at the Jos University Teaching hospital. *Nigerian Journal of Medicine*.

[110] Oniyangi O., Omari A. A. (2006). Malaria chemoprophylaxis in sickle cell disease. *Cochrane Database of Systematic Reviews*.

[111] Diop S., Soudré F., Seck M. (2011). Sickle-cell disease and malaria: evaluation of seasonal intermittent preventive treatment with sulfadoxine-pyrimethamine in Senegalese patients - a randomized placebo-controlled trial. *Annals of Hematology*.

[112] Olaosebikan R., Ernest K., Bojang K. (2015). A randomized trial to compare the safety, tolerability, and effectiveness of 3 antimalarial regimens for the prevention of malaria in Nigerian patients with sickle cell disease. *The Journal of Infectious Diseases*.

[113] Pickering L. K., Baker C. J., LS K. D. W., American Academy of Pediatrics (2012). *Red book: 2012 Report Of the Committee on Infectious Diseases*.

[114] Yanda A. N. A., Nansseu J. R. N., Awa H. D. M. (2017). Burden and spectrum of bacterial infections among sickle cell disease children living in Cameroon. *BMC Infectious Diseases*.

[115] Gaston M., Verter J., Woods G. (1986). Prophylaxis with oral penicillin in children with sickle cell anemia. *New England Journal of Medicine*.

[116] Williams T. N., Uyoga S., Macharia A. (2009). Bacteraemia in Kenyan children with sickle-cell anaemia: a retrospective cohort and case – control study. *The Lancet*.

[117] Kizito M. E., Mworozi E., Ndugwa C., Serjeant G. R. (2007). Bacteraemia in homozygous sickle cell disease in Africa: is pneumococcal prophylaxis justified?. *Archives Of Disease In Childhood*.

[118] Falletta J. M., Woods G. M., Verter J. I., Buchanan G. R., Pegelow C. H., Pegelow C. H. (1995). Discontinuing penicillin prophylaxis in children with sickle cell anemia. Prophylactic Penicillin Study II. *The Journal of Pediatrics*.

[119] Kateete D. P., Kajumbula H., Kaddu-Mulindwa D. H., Ssevviri A. K. (2012). Nasopharyngeal carriage rate of Streptococcus pneumoniae in Ugandan children with sickle cell disease. *BMC Research Notes*.

[120] Howard J. (2016). Sickle cell disease: when and how to transfuse. *Hematology 2014, the American Society of Hematology Education Program Book*.

[121] DeBaun M. (2016). The TWiTCH trial. *Hematology*.

[122] Adams R. J., Mckie V. C., Hsu L. (1998). Prevention of a first stroke by transfusions in children with sickle cell anemia and abnormal results on transcranial doppler ultrasonography. *New England Journal of Medicine*.

[123] The Optimizing Primary Stroke Prevention in Sickle Cell Anemia (STOP 2) Trial Investigators (2005). Discontinuing prophylactic transfusions used to prevent stroke in sickle cell disease. *New England Journal of Medicine*.

[124] Diaku-akinwumi I. N., Abubakar S. B., Adegoke S. A. (2016). Blood transfusion services for patients with sickle cell disease in Nigeria. *International Health*.

[125] Ministry of Health (2016). Uganda Clinical Guidelines 2016. http://library.health.go.ug/publications/guidelines/uganda-clinical-guidelines-2016.

[126] National Institutes of Health (2002). *The management of sickle cell disease*.

[127] Qureshi A., Kaya B., Pancham S. (2018). Guidelines for the use of hydroxycarbamide in children and adults with sickle cell disease A British Society for Haematology Guideline. *British Journal Of Haematology*.

[128] Vargas-Schaffer G. (2010). Is the WHO analgesic ladder still valid? Twenty-four years of experience. *Canadian Family Physician*.

[129] Brandow A. M., Carroll C. P., Creary S. (2020). American Society of Hematology 2020 guidelines for sickle cell disease: management of acute and chronic pain. *Blood Advances*.

[130] World Health Organisation (2015). WHO Model List of Essential Medicines. http://www.who.int/medicines/publications/essentialmedicines/en/.

[131] Nevitt S. J., Jones A. P., Howard J. (2017). Hydroxyurea (hydroxycarbamide) for sickle cell disease. *Cochrane Database of Systematic Reviews*.

[132] Tshilolo L., Tomlinson G., Williams T. N. (2019). Hydroxyurea for children with sickle cell anemia in Sub-Saharan Africa. *New England Journal of Medicine*.

[133] Ware R. E. (2010). How I treat How I use hydroxyurea to treat young patients with sickle cell anemia. *The Journal of the American Society of Hematology*.

[134] Olupot-olupot P., Wabwire H., Ndila C. (2020). Characterising demographics, knowledge, practices and clinical care among patients attending sickle cell disease clinics in Eastern Uganda. *Wellcome Open Research*.

[135] Mpalampa L., Ndugwa C. M., Ddungu H., Idro R. (2012). Foetal haemoglobin and disease severity in sickle cell anaemia patients in Kampala, Uganda. *BMC Blood Disord [Internet]*.

[136] Brandow A. M., Jirovec D. L., Panepinto J. A. (2011). Hydroxyurea in children with sickle cell disease: practice patterns and barriers to utilization. *American Journal of Hematology*.

[137] Ofakunrin A. O. D., Adekola K., Okpe E. S. (2019). Level of utilization and provider-related barriers to hydroxyurea use in the treatment of sickle cell disease in Jos, Nigeria. *Blood*.

[138] Brandow A. M., Panepinto J. A. (2010). Hydroxyurea use in sickle cell disease: the battle with low prescription rates, poor patient compliance and fears of toxicities. *Expert Review of Hematology*.

[139] Lubeck D., Agodoa I., Bhakta N. (2019). Estimated life expectancy and income of patients with sickle cell disease compared with those without sickle cell disease. *JAMA Network Open*.

[140] Agrawal R. K., Patel R. K., Shah V., Nainiwal L., Trivedi B. (2014). Hydroxyurea in sickle cell disease: drug review. *Indian Journal of Hematology and Blood Transfusion*.

[141] ClinicalTrials gov (2017). Decitabine for high-risk sickle cell disease [Internet]. NCT01375608.

[142] Steinberg M. H., Sebastiani P. (2012). Genetic modifiers of sickle cell disease. *American Journal of Hematology*.

[143] Steinberg M. H., Voskaridou E., Kutlar A. (2003). Concordant fetal hemoglobin response to hydroxyurea in siblings with sickle cell disease. *American Journal of Hematology*.

[144] Ware R. E., Eggleston B., Redding-Lallinger R. (2002). Predictors of fetal hemoglobin response in children with sickle cell anemia receiving hydroxyurea therapy. *Blood, The Journal of the American Society of Hematology*.

[145] Rees D. C. (2011). The rationale for using hydroxycarbamide in the treatment of sickle cell disease. *Haematologica*.

[146] Wang W. C., Ware R. E., Miller S. T. (2011). A multicenter randomised controlled trial of hydroxyurea (hydroxycarbamide) in very young children with sickle cell anaemia. *Lancet*.

[147] Charache S., Terrin M. L., Moore R. D. (1995). Effect of hydroxyurea on the frequency of painful crises in sickle cell anemia. *New England Journal of Medicine*.

[148] Ferster A., Vermylen C., Cornu G. (1996). Hydroxyurea for treatment of severe sickle cell anemia: a pediatric clinical trial. *Blood*.

[149] Hankins J. S., Ware R. E., Rogers Z. R. (2005). Long-term hydroxyurea therapy for infants with sickle cell anemia: the HUSOFT extension study. *Blood*.

[150] Platt O. S., Brambilla D. J., Rosse W. F. (1994). Mortality in sickle cell disease: Life expectancy and risk factors for early death. *New England Journal of Medicine*.

[151] Aygun B., Mortier N. A., Smeltzer M. P., Shulkin B. L., Hankins J. S., Ware R. E. (2013). Hydroxyurea treatment decreases glomerular hyperfiltration in children with sickle cell anemia. *American Journal of Hematology*.

[152] Ware R. E., Davis B. R., Schultz W. H. (2016). Hydroxycarbamide versus chronic transfusion for maintenance of transcranial doppler flow velocities in children with sickle cell anaemia — TCD With Transfusions Changing to Hydroxyurea (TWiTCH): a multicentre, open-label, phase 3, non-inferiority trial. *The Lancet*.

[153] Kinney T. R., Helms R. W., O’Branski E. E. (1999). Safety of hydroxyurea in children with sickle cell anemia: results of the HUG-KIDS Study, a Phase I/II trial. Pediatric Hydroxyurea Group. *Blood, The Journal of the American Society of Hematology*.

[154] Mvalo T., Topazian H. M., Kamthunzi P. (2019). Real-world experience using hydroxyurea in children with sickle cell disease in Lilongwe, Malawi. *Pediatric Blood & Cancer*.

[155] Ware R. E., Aygun B. (2009). Advances in the use of hydroxyurea. *ASH Education Program Book*.

[156] Schultz W. H., Ware R. E. (2003). Malignancy in patients with sickle cell disease. *American Journal of Hematology*.

[157] Branski E. E. O., Ware R. E., Prose N. S., Kinney T. R., Carolina N. (2001). Skin and nail changes in children with sickle cell anemia receiving hydroxyurea therapy. *Journal of the American Academy of Dermatology*.

[158] Aste N., Fumo G., Contu F., Aste N., Biggio P. (2002). Nail pigmentation caused by hydroxyurea: report of 9 cases. *Journal of the American Academy of Dermatology*.

[159] Obaro S. K. (2015). Hydroxyurea for sickle-cell anaemia in Africa: mind the gap. *The Lancet Global Health*.

[160] Coache D., Friciu M., Bernine Marcellin R. (2022). Stability evaluation of compounded hydroxyurea 100 mg/mL oral liquids using a novel analytical method involving chemical derivatization. *PLoS One*.

[161] Ofakunrin A. O., Oguche S., Adekola K. (2020). Effectiveness and safety of hydroxyurea in the treatment of sickle cell anaemia children in Jos, North Central Nigeria. *Journal of Tropical Pediatrics*.

[162] Creary S. E., Strouse J. J. (2014). Hydroxyurea and transfusion therapy sickle cell disease. https://www.hematology.org/-/media/hematology/files/education/clinicians/guidelines-quality/documents/sickle-cell-hydroxyurea.pdf.

[163] Sadaf A., Quinn C. T. (2020). L-glutamine for sickle cell disease: knight or pawn?. *Experimental Biology and Medicine*.

[164] Food and Drug Administration (2017). FDA approves new treatment for sickle cell disease. https://www.fda.gov/news-events/press-announcements/fda-approves-new-treatment-sickle-cell-disease.

[165] Niihara Y., Macan H., Eckman J. R. (2014). L-glutamine therapy reduces hospitalization for sickle cell anemia and Sickle *β*°-thalassemia patients at six months – a phase II randomized trial. *Clinical Pharmacology and Biopharmaceutics*.

[166] Niihara Y., Miller S. T., Kanter J. (2018). A phase 3 trial of L-Glutamine in sickle cell disease. *New England Journal of Medicine*.

[167] ClinicalTrials gov (2020). Pharmacokinetics and safety of Endari (L-glutamine) in sickle cell disease patients. NCT04684381.

[168] Riley T. R., Boss A., Mcclain D., Riley T. T. (2018). Review of medication therapy for the prevention of sickle cell crisis. *Pharmacy and Therapeutics*.

[169] Quinn C. T. (2018). L-Glutamine for sickle cell anemia: more questions than answers. *Blood*.

[170] Ataga K. I., Kutlar A., Kanter J. (2017). Crizanlizumab for the prevention of pain crises in sickle cell disease. *New England Journal of Medicine*.

[171] ClinicalTrials gov (2022). Study of dose confirmation and safety of Crizanlizumab in pediatric sickle cell disease Patients. https://clinicaltrials.gov/ct2/show/NCT03474965.

[172] Hutchaleelaha A., Patel M., Washington C. (2019). Pharmacokinetics and pharmacodynamics of voxelotor (GBT440) in healthy adults and patients with sickle cell disease. *British Journal of Clinical Pharmacology*.

[173] FDA (2022). FDA approves voxelotor for sickle cell disease. https://www.fda.gov/drugs/resources-information-approved-drugs/fda-approves-voxelotor-sickle-cell-disease.

[174] FDA (2022). FDA approves drug to treat sickle cell disease in patients aged 4 up to 11 years. https://www.fda.gov/drugs/resources-information-approved-drugs/fda-approves-voxelotor-sickle-cell-disease.

[175] Global Blood Therapeutics (2022). European Commission approves Oxbryta ® (voxelotor) for the treatment of hemolytic anemia in patients with sickle cell disease age 12 years and older. https://ir.gbt.com/news-releases/news-release-details/european-commission-approves-oxbrytar-voxelotor-treatment.

[176] Vichinsky E., Hoppe C. C., Ataga K. I. (2019). A phase 3 randomized trial of Voxelotor in sickle cell disease. *New England Journal of Medicine*.

[177] Estepp J. H., Kalpatthi R., Woods G. (2022). Safety and efficacy of voxelotor in pediatric patients with sickle cell disease aged 4 to 11 years. *Pediatric Blood & Cancer*.

[178] ClinicalTrials gov (2021). Oxbryta® Product Registry an observational study designed to evaluate the effect of Oxbryta in individuals with SCD (PROSPECT). NCT04930445.

[179] ClinicalTrials gov (2021). Study to evaluate the effect of GBT440 on TCD in pediatrics with sickle cell disease (HOPE Kids 2). NCT04218084.

[180] Johnson F. L., Look A. T., Gockerman J., Ruggiero M. R., Dalla-Pozza L., Billings F. T. (1984). Bone-marrow transplantation in a patient with sickle-cell anemia. *New England Journal of Medicine*.

[181] Gluckman E., Cappelli B., Bernaudin F. (2017). Sickle cell disease: an international survey of results of HLA-identical sibling hematopoietic stem cell transplantation. *Blood*.

[182] Aydin M., Dovern E., Lee M. M. G. (2021). Haploidentical allogeneic stem cell transplantation in sickle cell disease: a systematic review and meta-analysis. *Transplantation and Cellular Therapy*.

[183] Harif M., Weisdorf D., Novitzky N., Szer J. (2019). Special report: summary of the first meeting of African Blood and Marrow Transplantation (AfBMT) group, Casablanca, Morocco, April 19–21, 2018 held under the auspices of the Worldwide Network for Blood and Marrow Transplantation (WBMT). *Hematology/Oncology and Stem Cell Therapy*.

[184] Mtenga J., Orf K., Zheng J. (2021). Haematopoietic stem cell transplantation in Tanzania. *British Journal of Haematology*.

[185] Makani J. (2020). Curative options for sickle cell disease in Africa: approach in Tanzania. *Hematology/Oncology and Stem Cell Therapy*.

[186] Daak A. A., Lopez-toledano M. A., Heeney M. M. (2020). Biochemical and therapeutic effects of Omega-3 fatty acids in sickle cell disease. *Complementary Therapies in Medicine*.

[187] Calder P. C. (2014). Marine omega-3 fatty acids and inflammatory processes: effects, mechanisms and clinical relevance. *Biochimica et Biophysica Acta (BBA)-Molecular and Cell Biology of Lipids*.

[188] Calder P. C. (2010). Omega-3 fatty acids and inflammatory processes. *Nutrients*.

[189] Aslan M., Celmeli G., Ozcan F., Kupesiz A. (2015). LC-MS/MS analysis of plasma polyunsaturated fatty acids in patients with homozygous sickle cell disease. *Clinical and Experimental Medicine*.

[190] Setty B. Y., Betal S. G., Miller R. E. (2019). Relationship of omega-3 fatty acids DHA and EPA with the inflammatory biomarker hs-CRP in children with sickle cell anemia. *Prostaglandins, Leukotrienes and Essential Fatty Acids*.

[191] Tomer A., Kasey S., Connor W. E., Clark S., Harker L. A., Eckman J. R. (2001). Reduction of pain episodes and prothrombotic activity in sickle cell disease by dietary n-3 fatty acids. *Thrombosis and Haemostasis*.

[192] Daak A. A., Ghebremeskel K., Hassan Z. (2013). Effect of omega-3 (n-3) fatty acid supplementation in patients with sickle cell anemia: randomized, double-blind, placebo-controlled. *The American Journal of Clinical Nutrition*.

[193] Demirci S., Uchida N., Tisdale J. F. (2018). Gene therapy for sickle cell disease: an update. *Cytotherapy*.

[194] Kanter J., Falcon C. (2021). Gene therapy for sickle cell disease: where we are now?. *Hematology*.

[195] Kanter J., Walters M. C., Krishnamurti L. (2022). Biologic and clinical efficacy of LentiGlobin for sickle cell disease. *New England Journal of Medicine*.

[196] Esrick E. B., Lehmann L. E., Biffi A. (2021). Post-transcriptional genetic silencing of BCL11A to treat sickle cell disease. *New England Journal of Medicine*.

[197] Magrin E., Magnani A., Semeraro M. (2021). Clinical results of the Drepaglobe trial for sickle cell disease patients. *Blood*.

[198] Alavi A., Krishnamurti L., Abedi M. (2021). Preliminary safety and efficacy results from Precizn-1: an ongoing Phase 1/2 study on Zinc Finger Nuclease-Modified Autologous CD34+ HSPCs for sickle cell disease (SCD). *Blood*.

[199] ClinicalTrials gov (2022). Gene transfer for patients with sickle cell disease. https://www.clinicaltrials.gov/ct2/show/NCT02186418.

[200] ClinicalTrials gov (2022). Gene correction in autologous CD34+ hematopoietic stem cells (HbS to HbA) to treat severe sickle cell disease (CEDAR). NCT04819841.

[201] Frangoul H., Altshuler D., Cappellini M. D. (2021). CRISPR-Cas 9 gene editing for sickle cell disease and *β*-thalassemia. *New England Journal of Medicine*.

[202] Kayle M., Docherty S. L., Sloane R. (2018). Transition to adult care in sickle cell diseas: a longitudinal study of clinical characteristics and disease severity. *Pediatric Blood & Cancer*.

[203] Hankins J. S., Osarogiagbon R., Adams-graves P. (2012). A transition pilot program for adolescents with sickle cell disease. *Journal of Pediatric Health Care*.

[204] Hassell K. L. (2010). Population estimates of sickle cell disease in the U.S. *American Journal of Preventive Medicine*.

[205] Kanter J., Gibson R., Lawrence R. H. (2020). Perceptions of US adolescents and adults with sickle cell disease on their quality of care. *JAMA Network Open*.

[206] Gray W. N., Schaefer M. R., Resmini-rawlinson A., Wagoner S. T. (2018). Barriers to transition from pediatric to adult care: a systematic review. *Journal of Pediatric Psychology*.

[207] Lanzkron S., Sawicki G. S., Hassell K. L., Konstan M. W., Liem R. I., Mccolley S. A. (2018). Transition to adulthood and adult health care for patients with sickle cell disease or cystic fibrosis: current practices and research priorities. *Journal of Clinical and Translational Science*.

[208] Saulsberry A. C., Porter J. S., Hankins J. S. (2019). A program of transition to adult care for sickle cell disease. *Hematology 2014, the American Society of Hematology Education Program Book*.

[209] Mulchan S. S., Valenzuela J. M., Crosby L. E., Sang C. D. P. (2016). Applicability of the SMART Model of transition readiness for sickle-cell disease. *Journal of Pediatric Psychology*.

